# Supplementary figures and images for: Covalent inhibition of endoplasmic reticulum chaperone GRP78 disconnects the transduction of ER stress signals to inflammation and lipid accumulation in diet-induced obese mice
Source: eLife. 2022 Feb 9;11:e72182. doi: 10.7554/eLife.72182 (PMC8828050; doi:10.7554/eLife.72182)

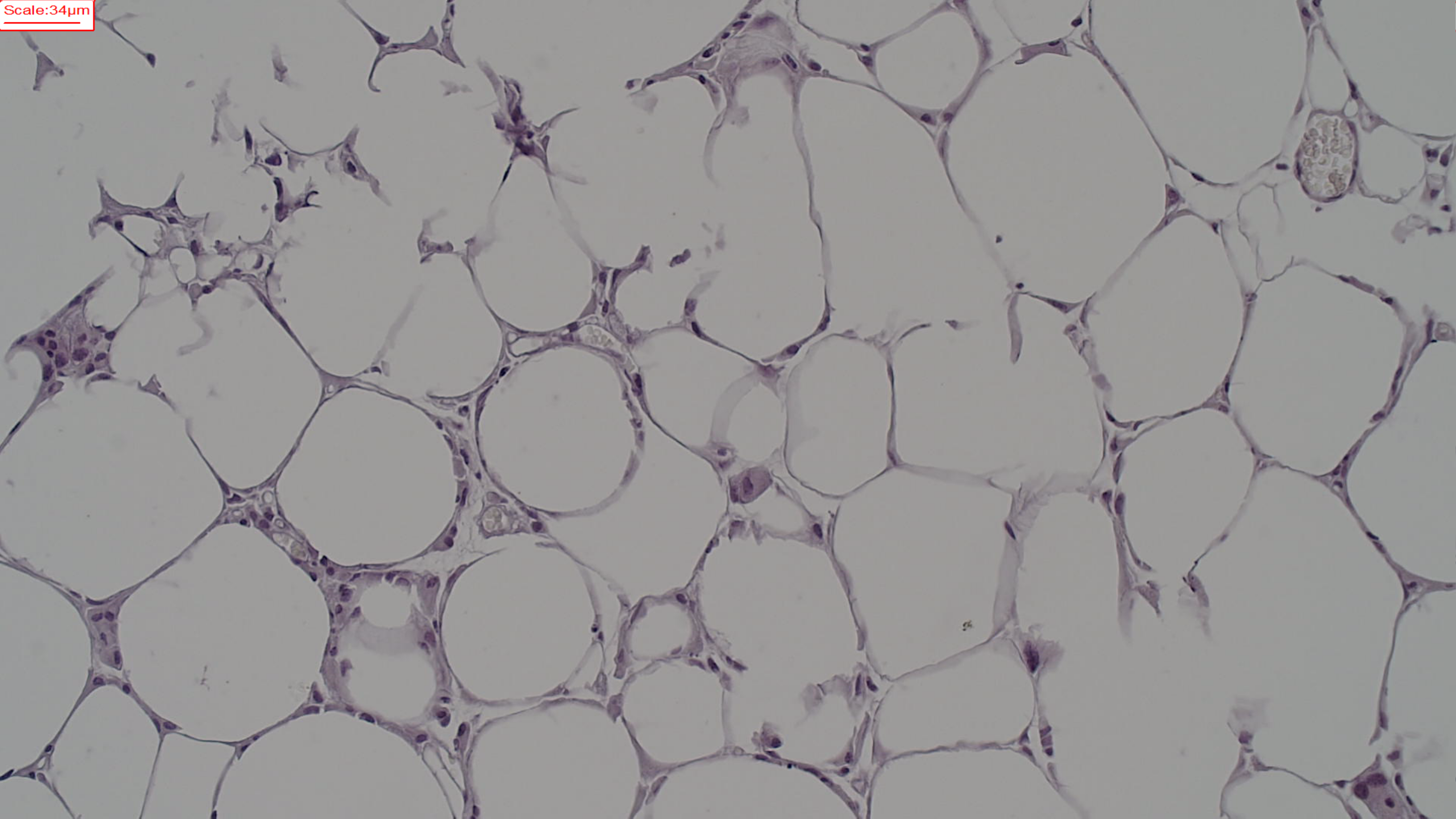

Supplement: Figure 1—source data 1. [file elife-72182-fig1-data1.zip › Figure 1-source data 1/Control-Adipose tissue.tif]

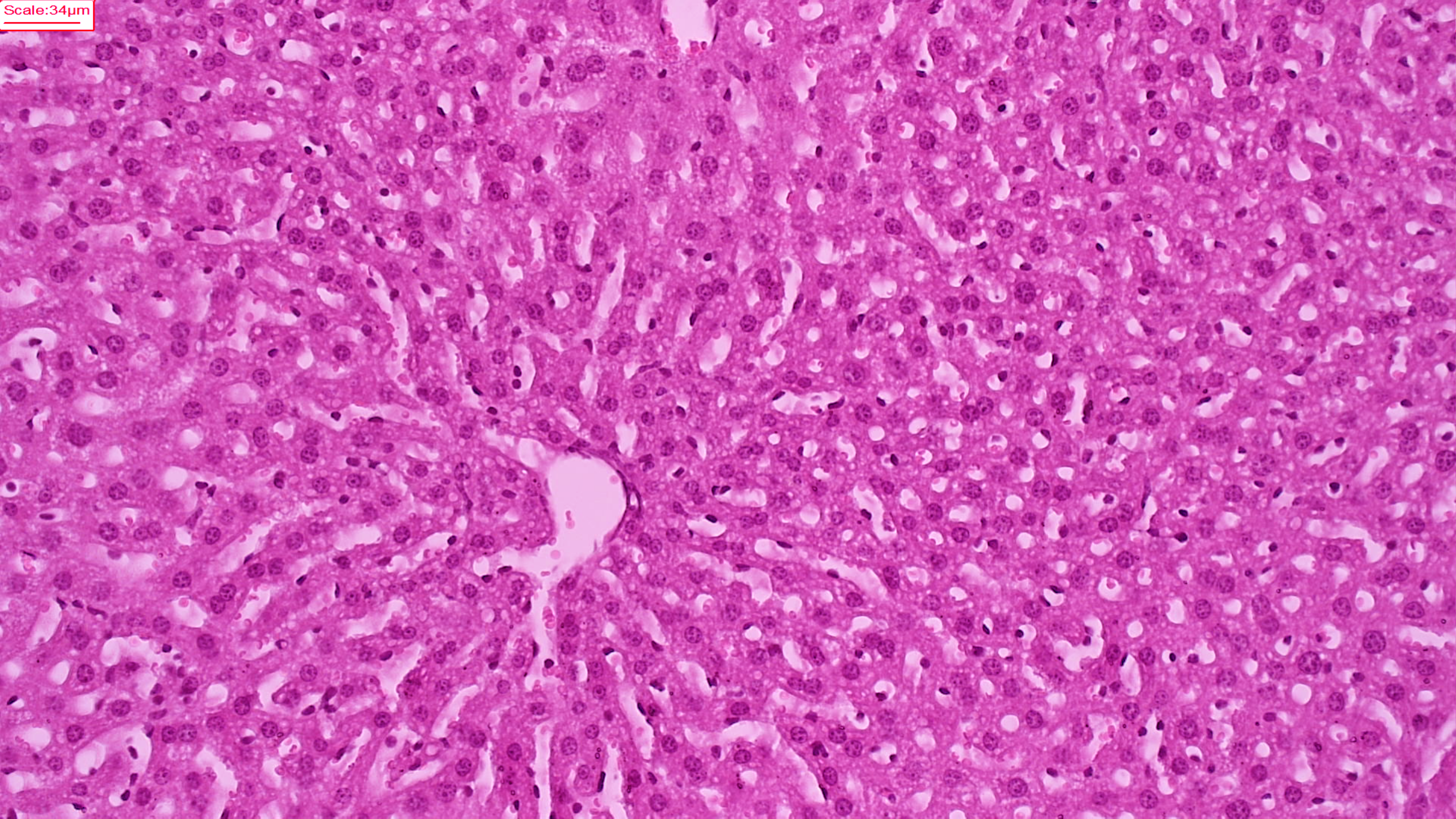

Supplement: Figure 1—source data 1. [file elife-72182-fig1-data1.zip › Figure 1-source data 1/Control-Liver.tif]

## Slide 1
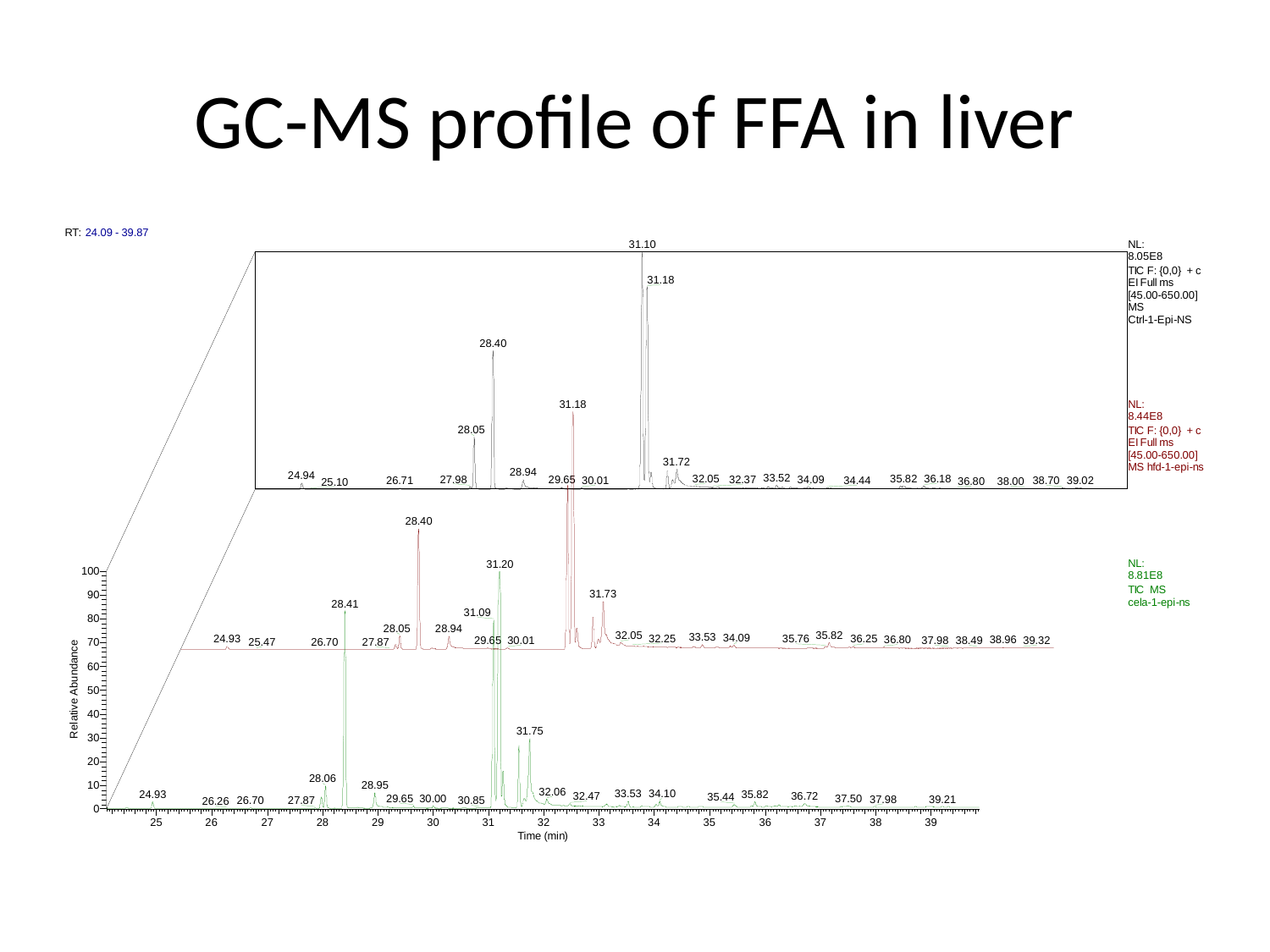

# GC-MS profile of FFA in liver

## Slide 2
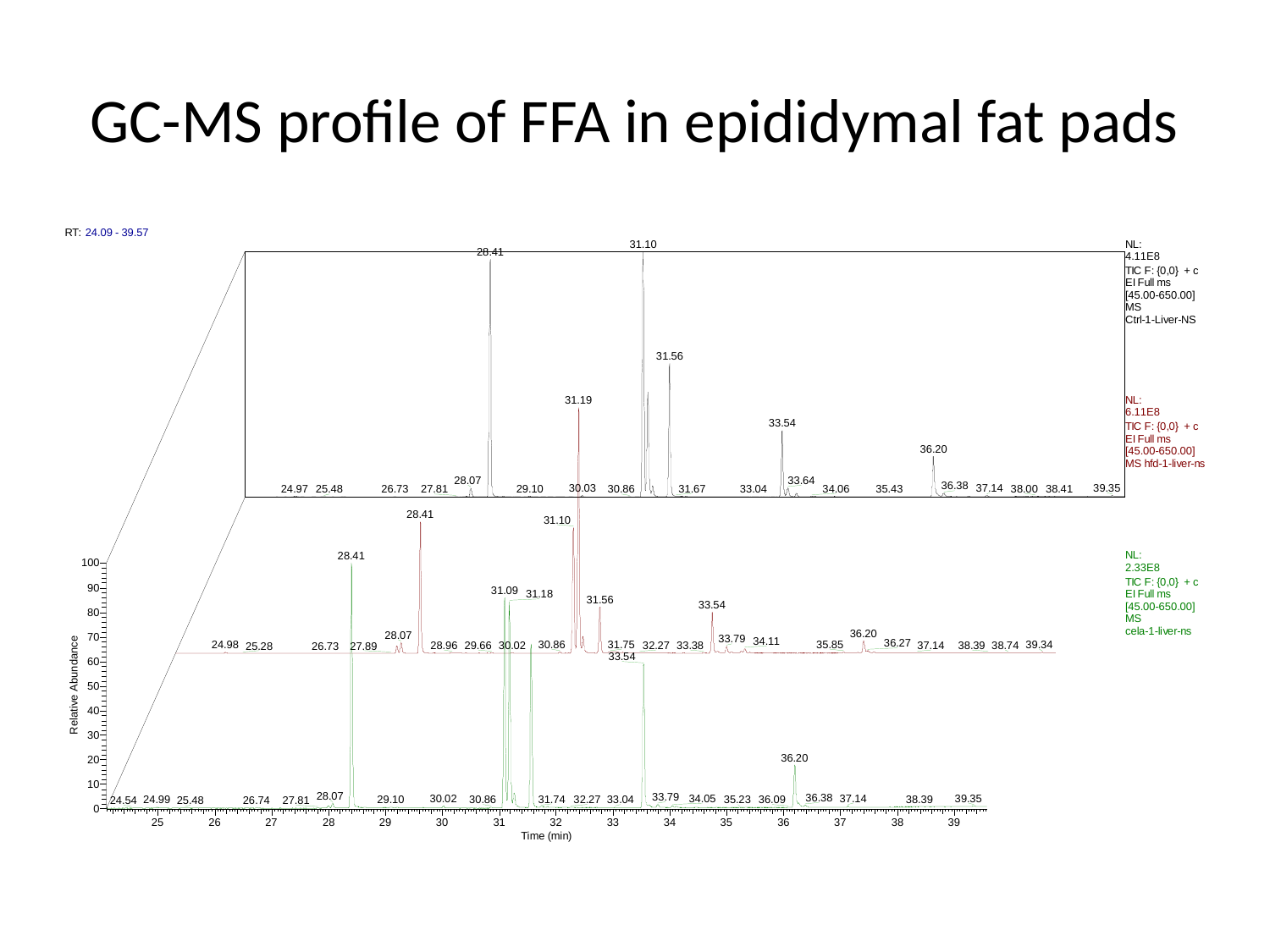

# GC-MS profile of FFA in epididymal fat pads

Supplement: Figure 1—source data 1. [file elife-72182-fig1-data1.zip › Figure 1-source data 1/Figure 1C-1D.pptx]

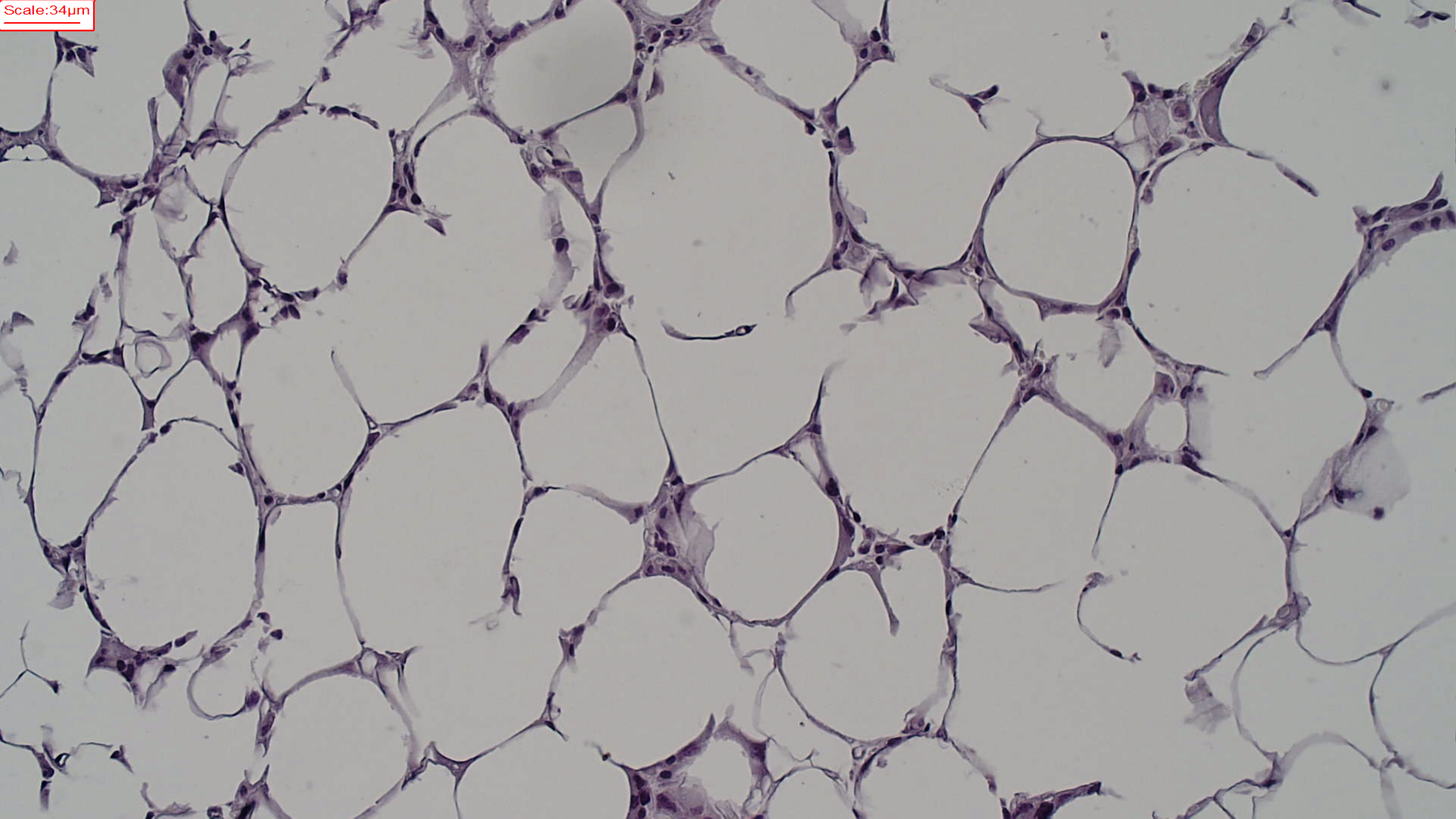

Supplement: Figure 1—source data 1. [file elife-72182-fig1-data1.zip › Figure 1-source data 1/HFD+Celastrol-adipose tissue.tif]

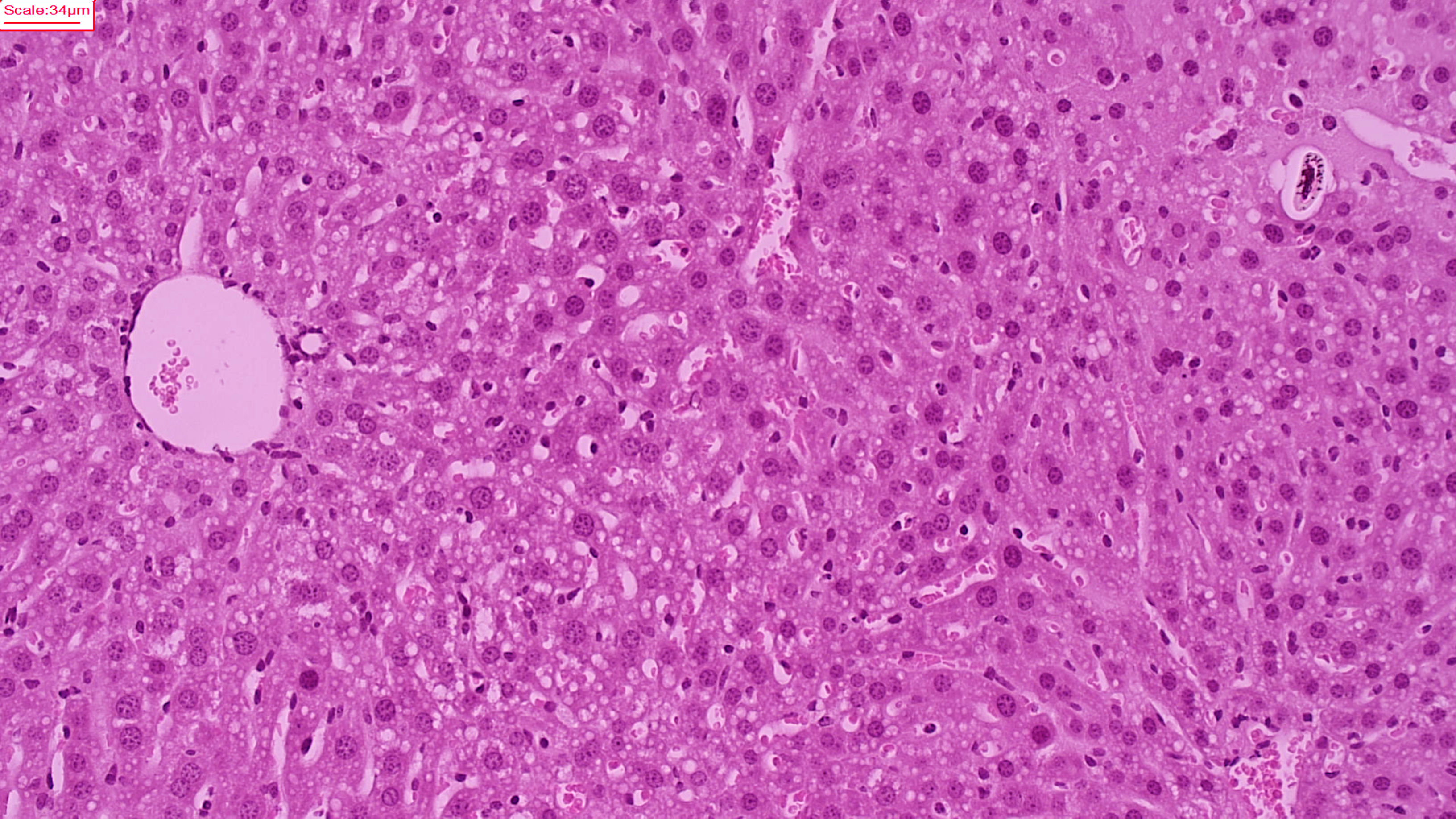

Supplement: Figure 1—source data 1. [file elife-72182-fig1-data1.zip › Figure 1-source data 1/HFD+Celastrol-liver.tif]

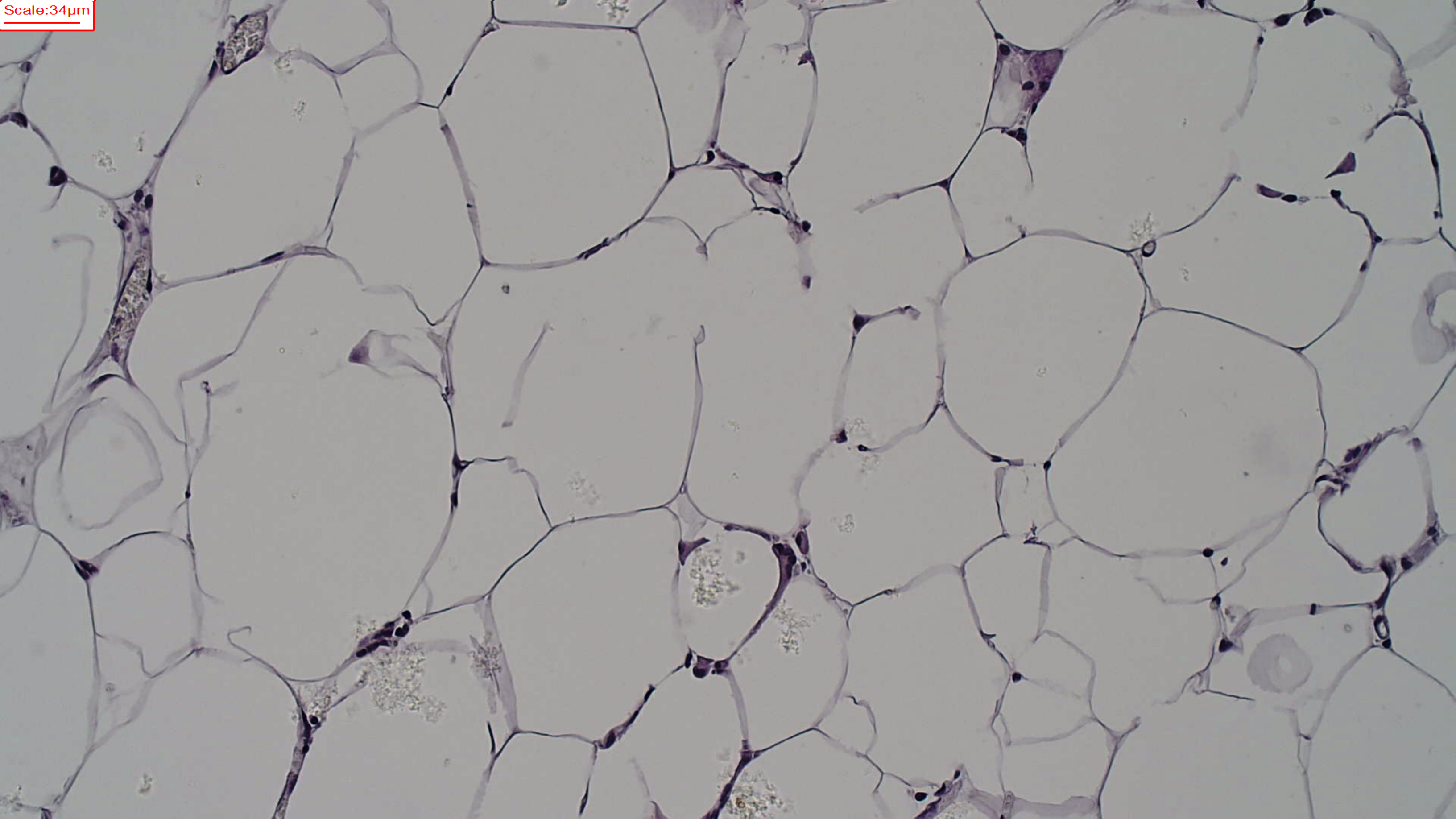

Supplement: Figure 1—source data 1. [file elife-72182-fig1-data1.zip › Figure 1-source data 1/HFD-Adipose tissue.tif]

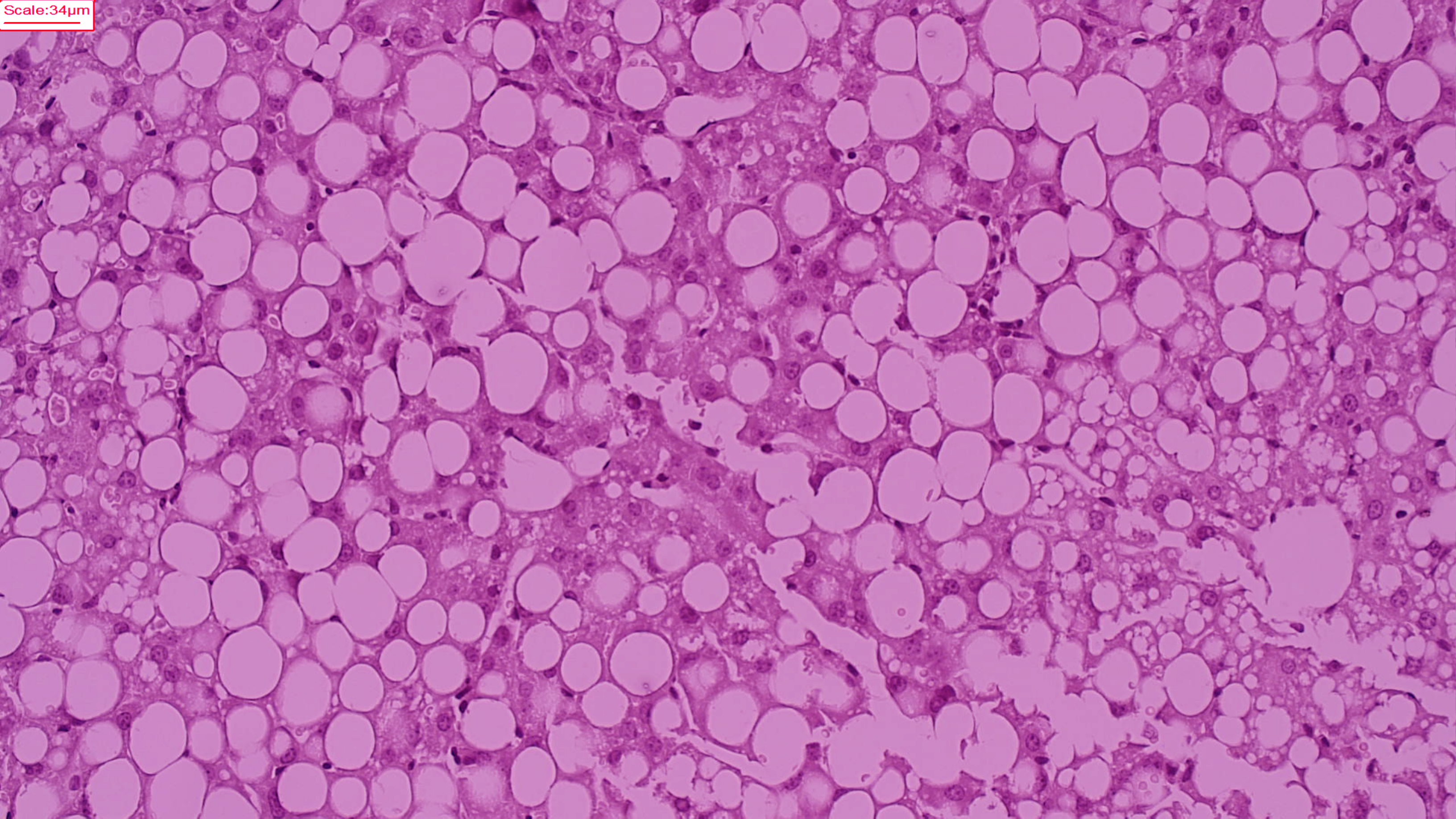

Supplement: Figure 1—source data 1. [file elife-72182-fig1-data1.zip › Figure 1-source data 1/HFD-Liver.tif]

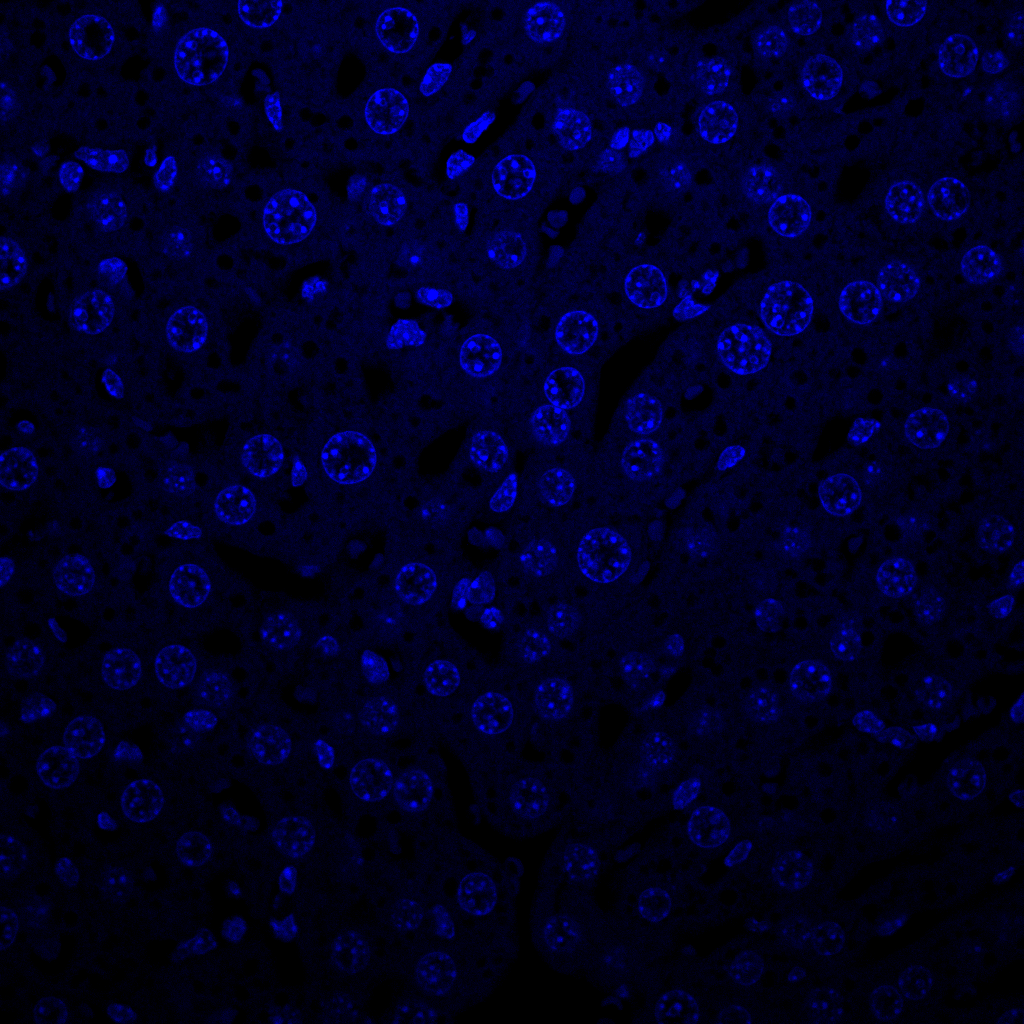

Supplement: Figure 3—source data 1. [file elife-72182-fig3-data1.zip › Liver/cela bip liver 1(dapi).tif]

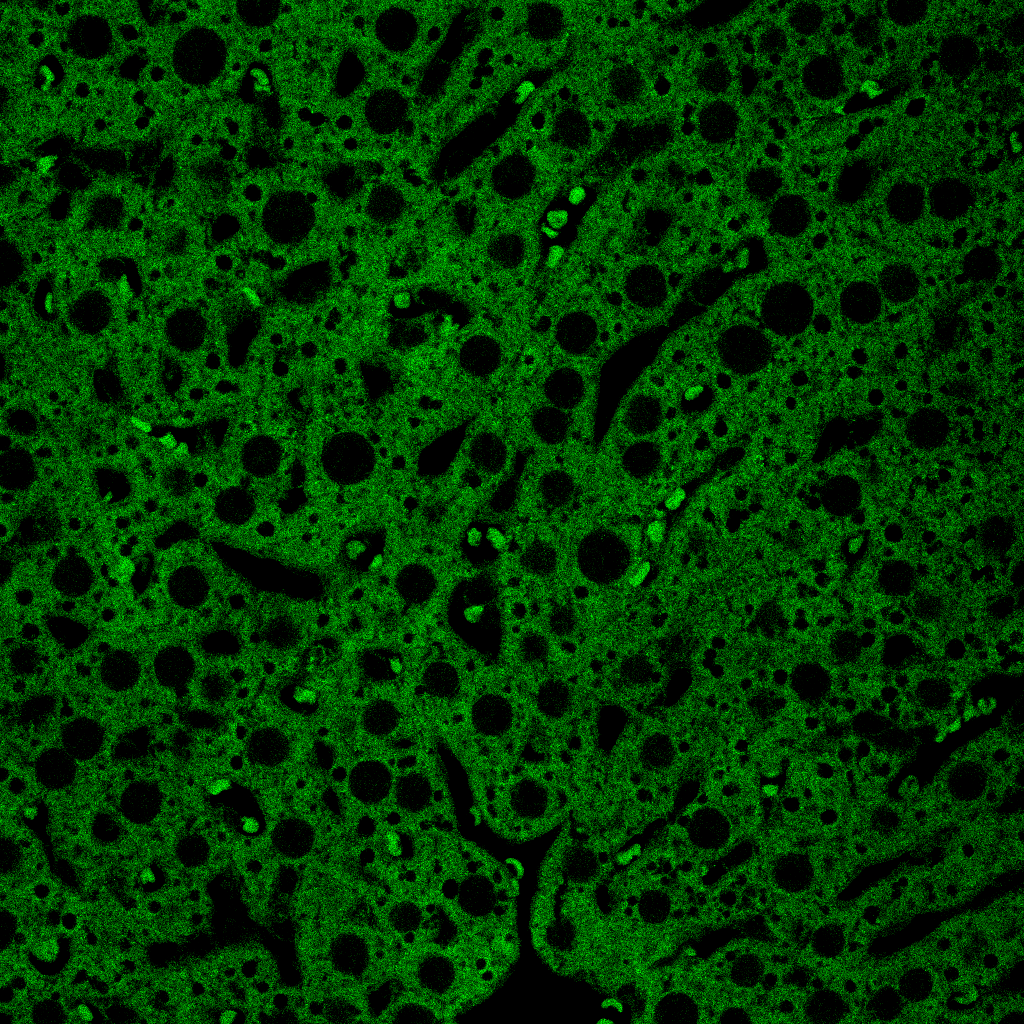

Supplement: Figure 3—source data 1. [file elife-72182-fig3-data1.zip › Liver/cela bip liver 1(green).tif]

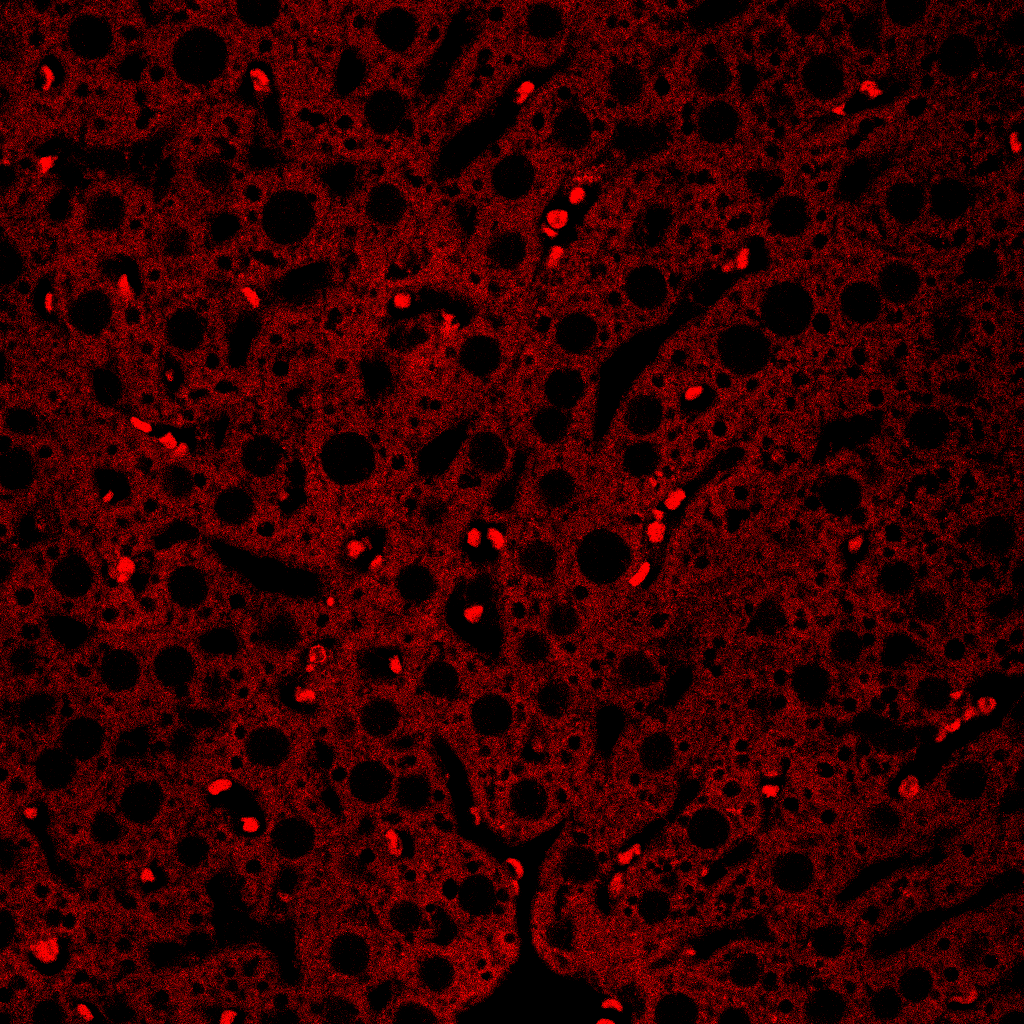

Supplement: Figure 3—source data 1. [file elife-72182-fig3-data1.zip › Liver/cela bip liver 1(red).tif]

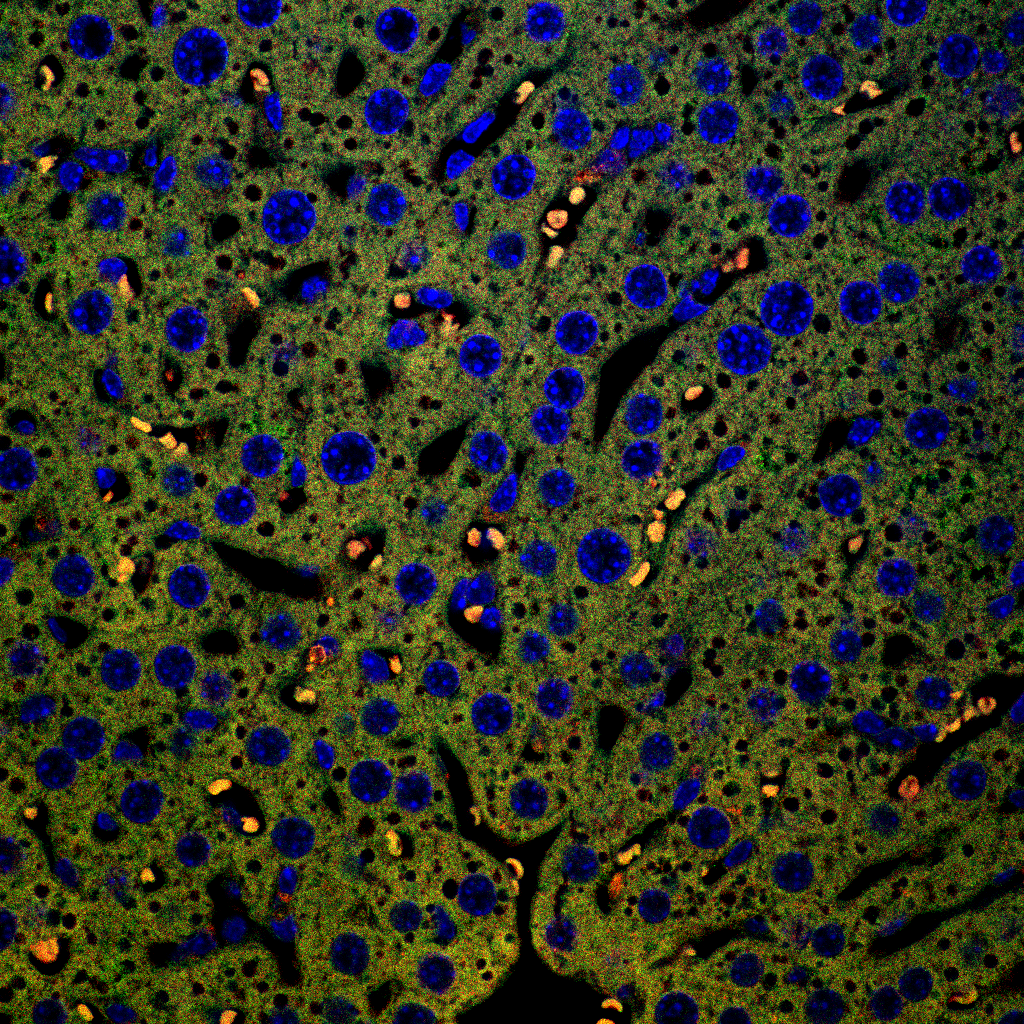

Supplement: Figure 3—source data 1. [file elife-72182-fig3-data1.zip › Liver/cela bip liver 1.tif]

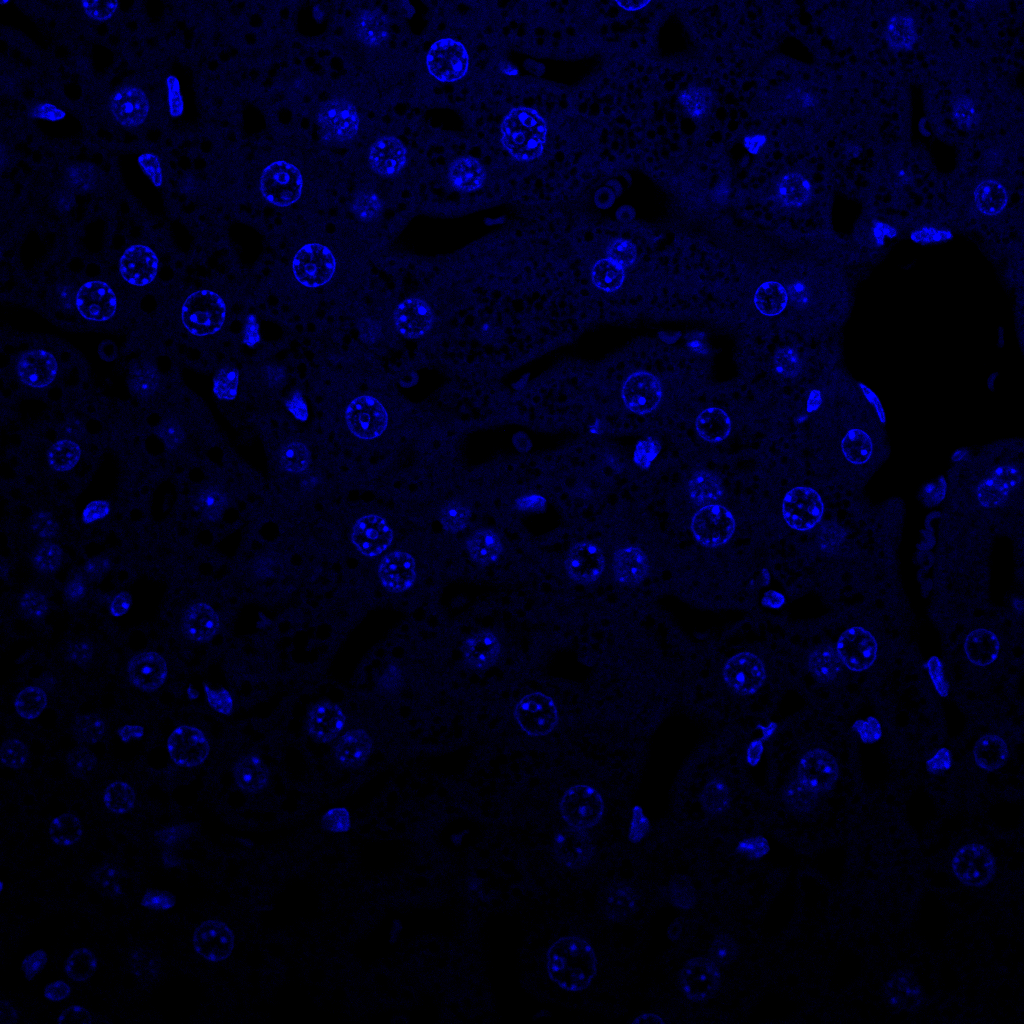

Supplement: Figure 3—source data 1. [file elife-72182-fig3-data1.zip › Liver/ctrl bip liver (dapi).tif]

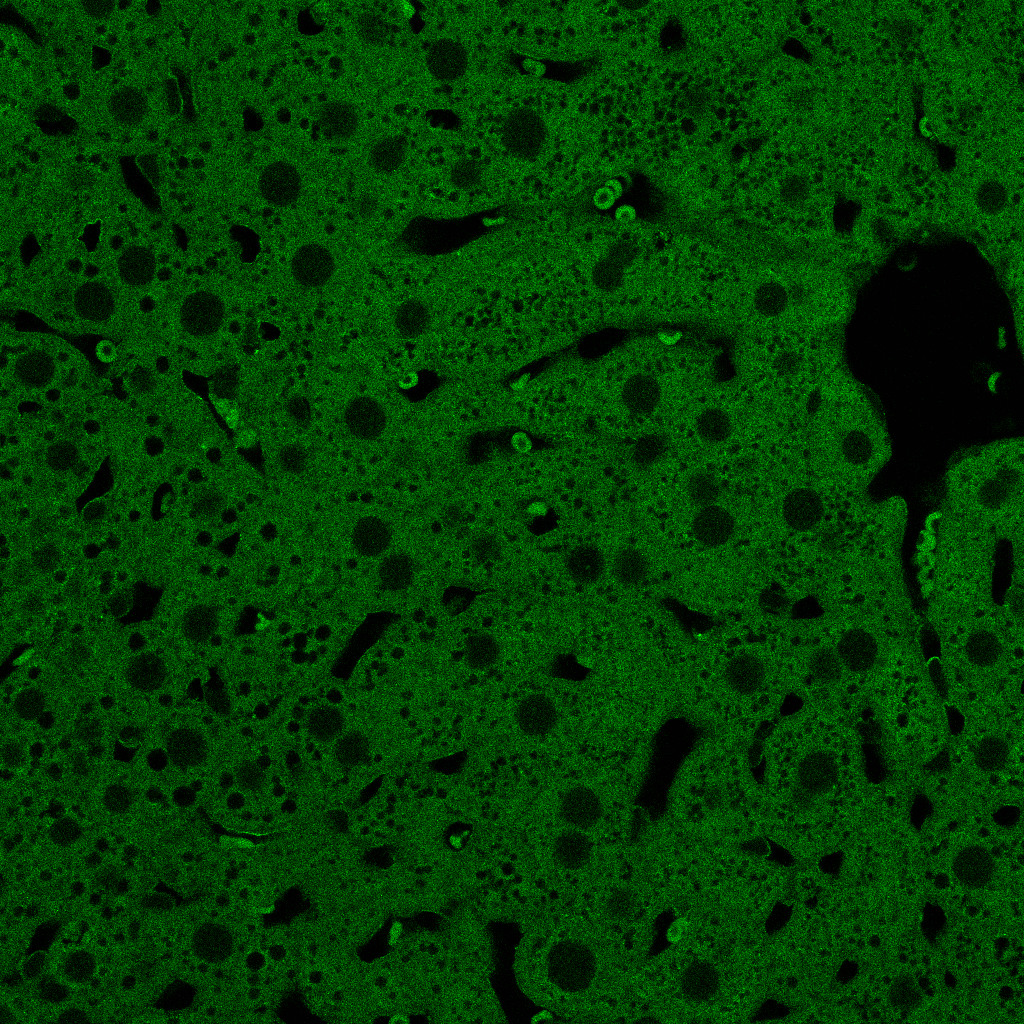

Supplement: Figure 3—source data 1. [file elife-72182-fig3-data1.zip › Liver/ctrl bip liver (green).tif]

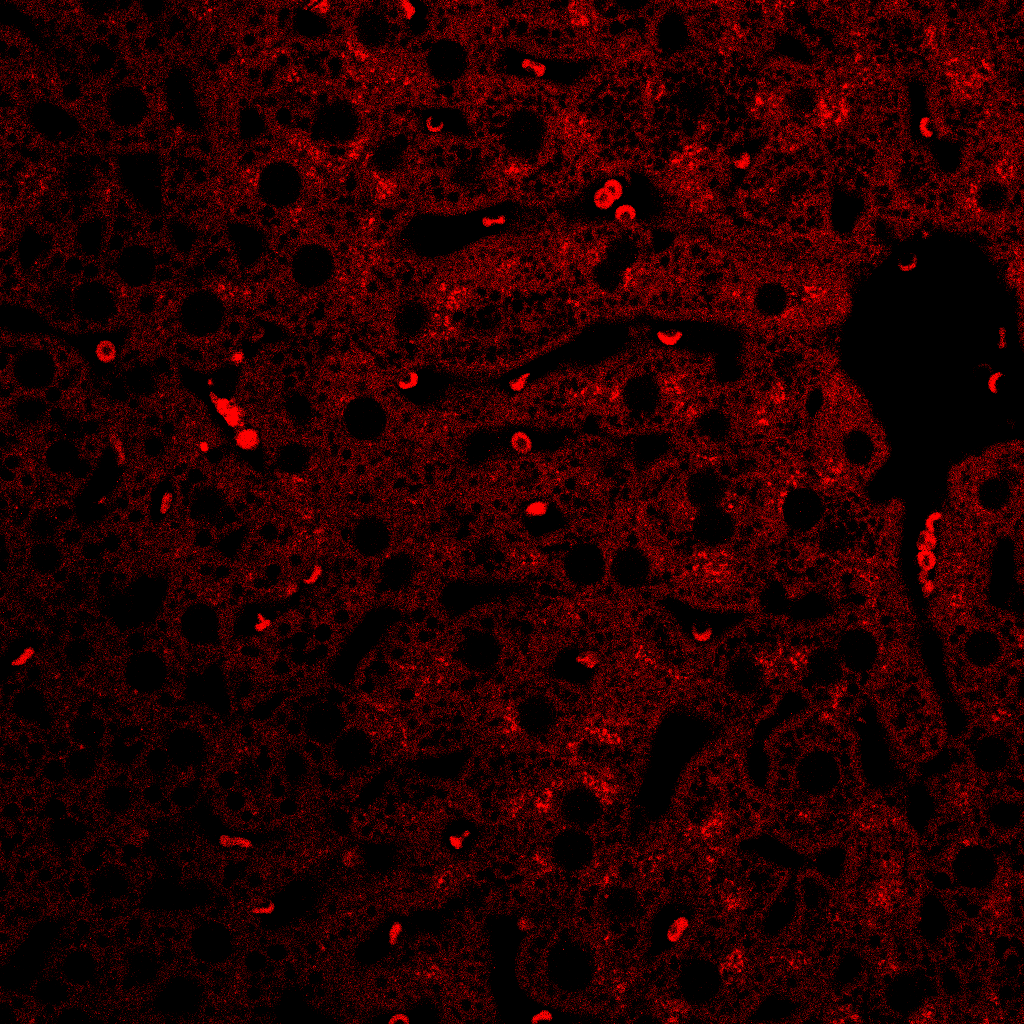

Supplement: Figure 3—source data 1. [file elife-72182-fig3-data1.zip › Liver/ctrl bip liver (red).tif]

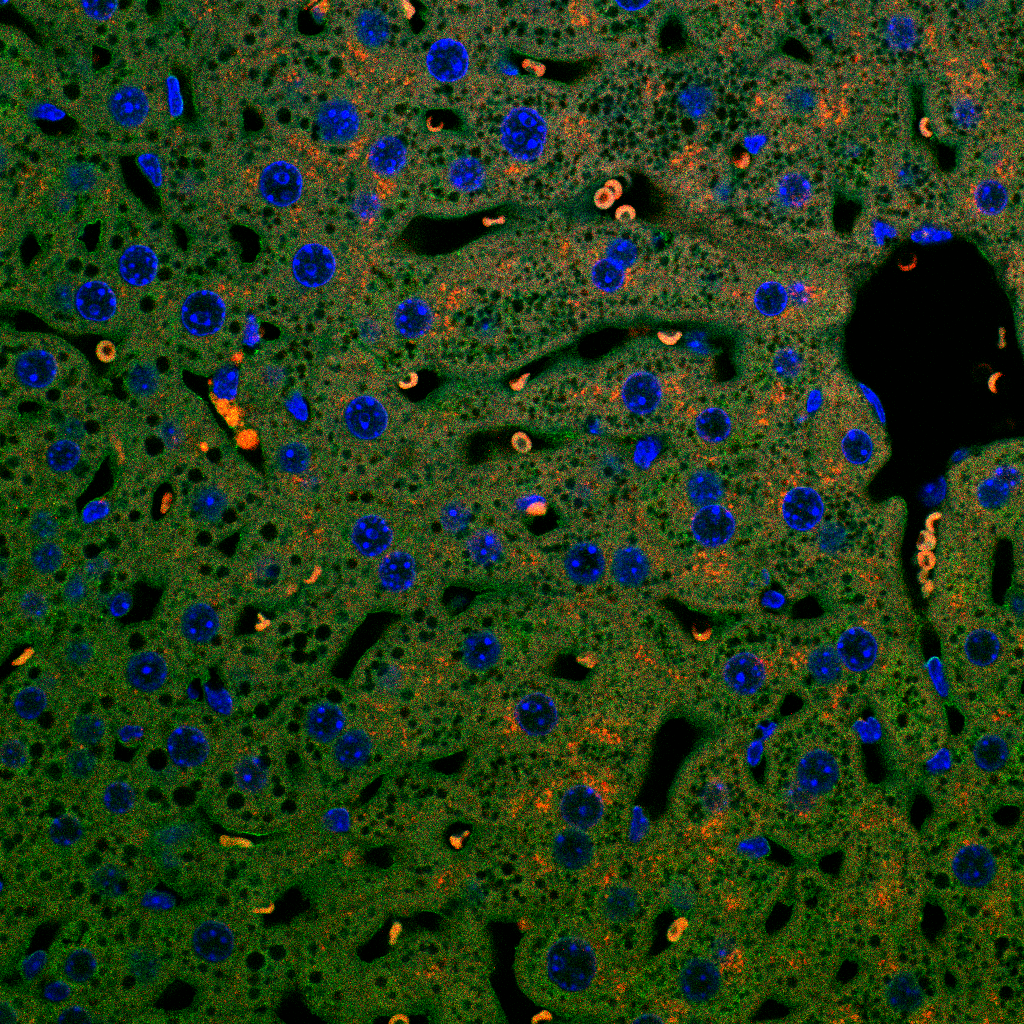

Supplement: Figure 3—source data 1. [file elife-72182-fig3-data1.zip › Liver/ctrl bip liver 1.tif]

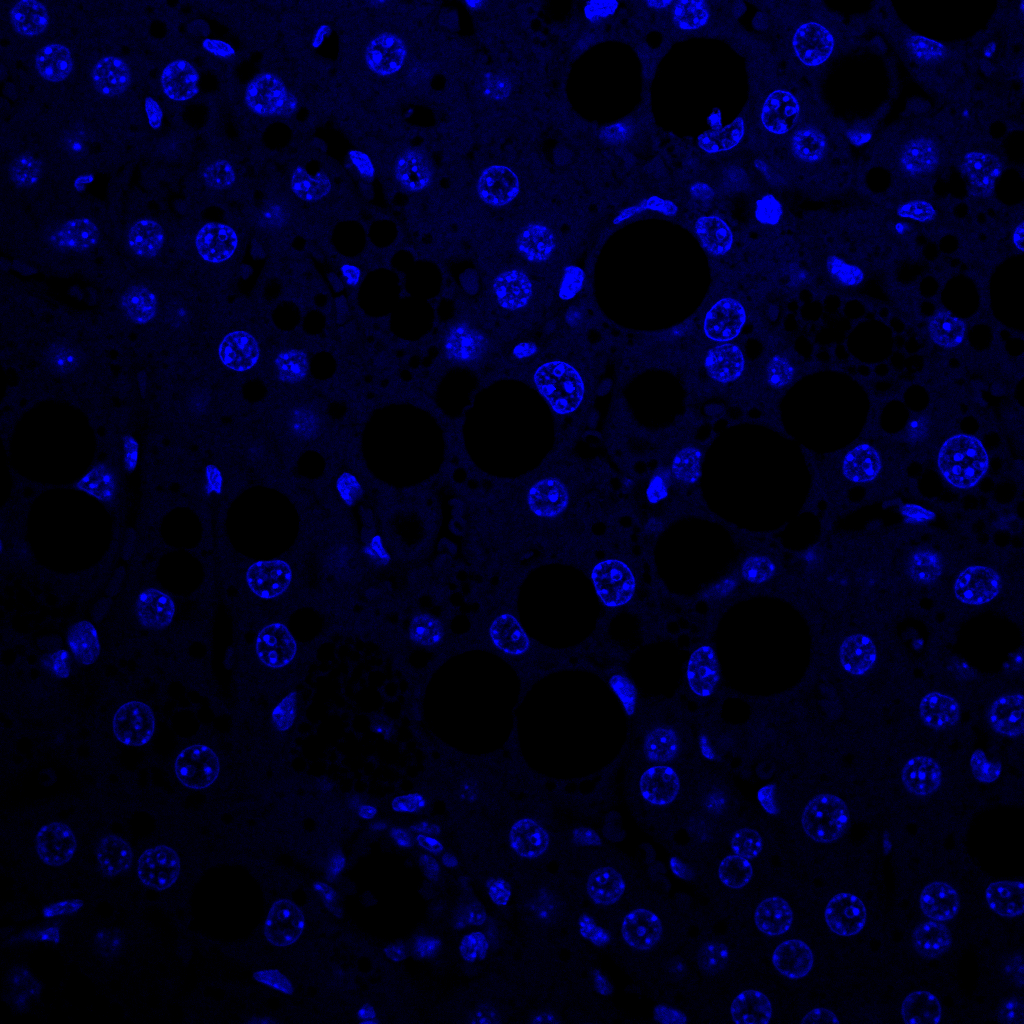

Supplement: Figure 3—source data 1. [file elife-72182-fig3-data1.zip › Liver/hfd bip GRP 1(dapi).tif]

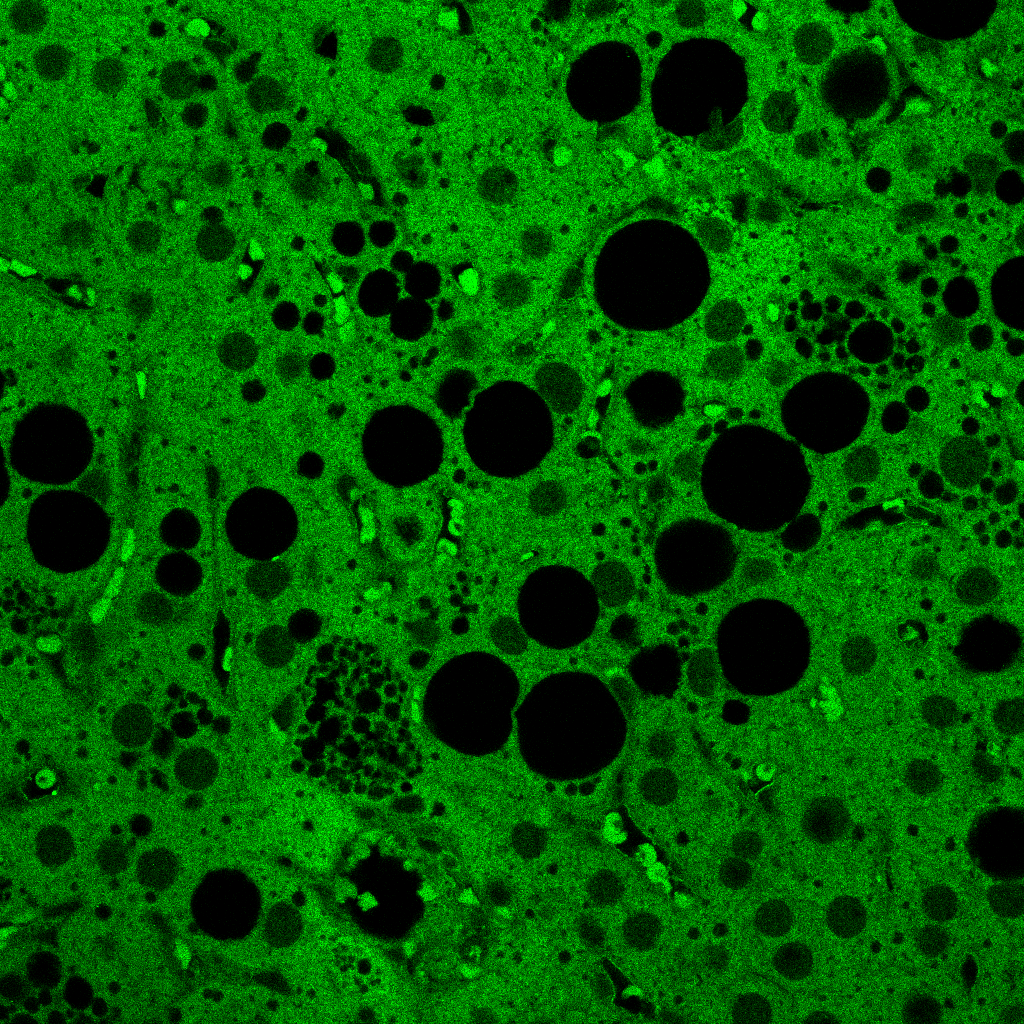

Supplement: Figure 3—source data 1. [file elife-72182-fig3-data1.zip › Liver/hfd bip GRP 1(green).tif]

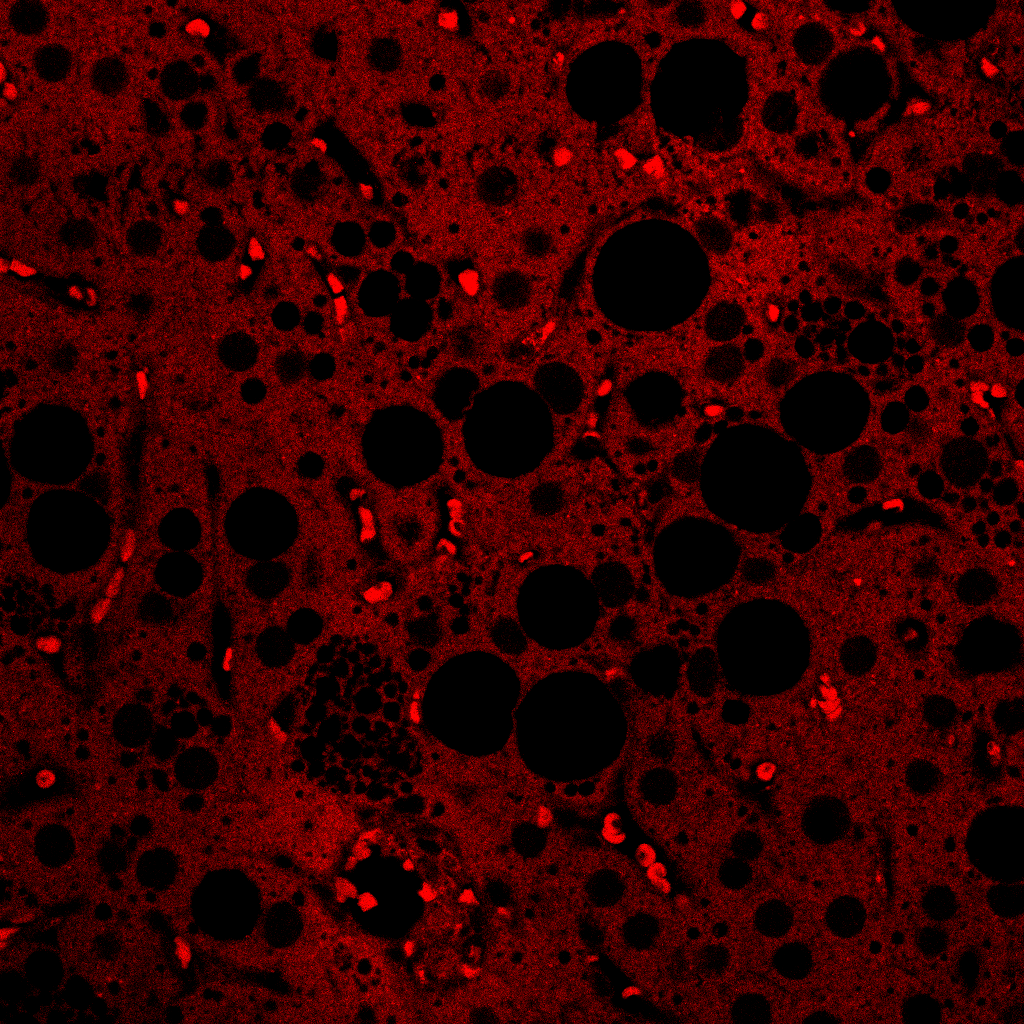

Supplement: Figure 3—source data 1. [file elife-72182-fig3-data1.zip › Liver/hfd bip GRP 1(red).tif]

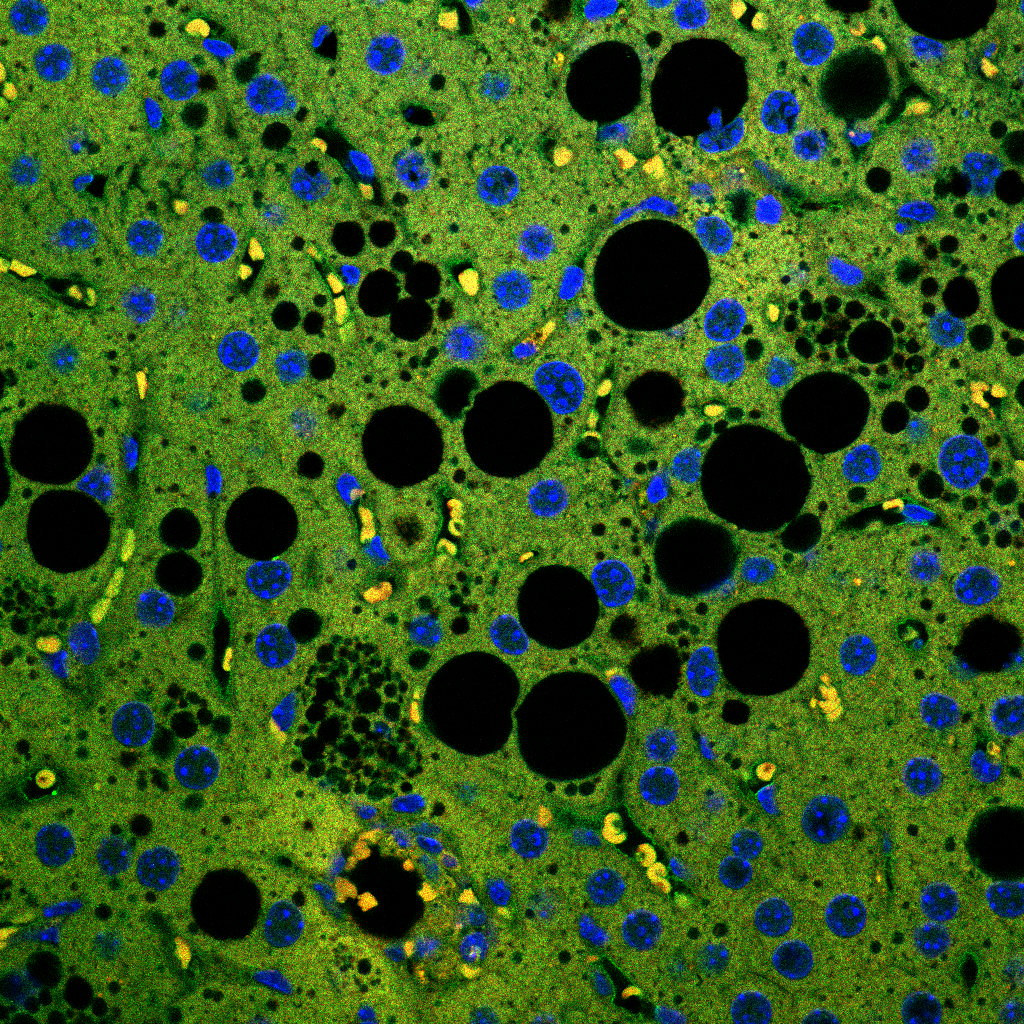

Supplement: Figure 3—source data 1. [file elife-72182-fig3-data1.zip › Liver/hfd bip GRP 1.tif]

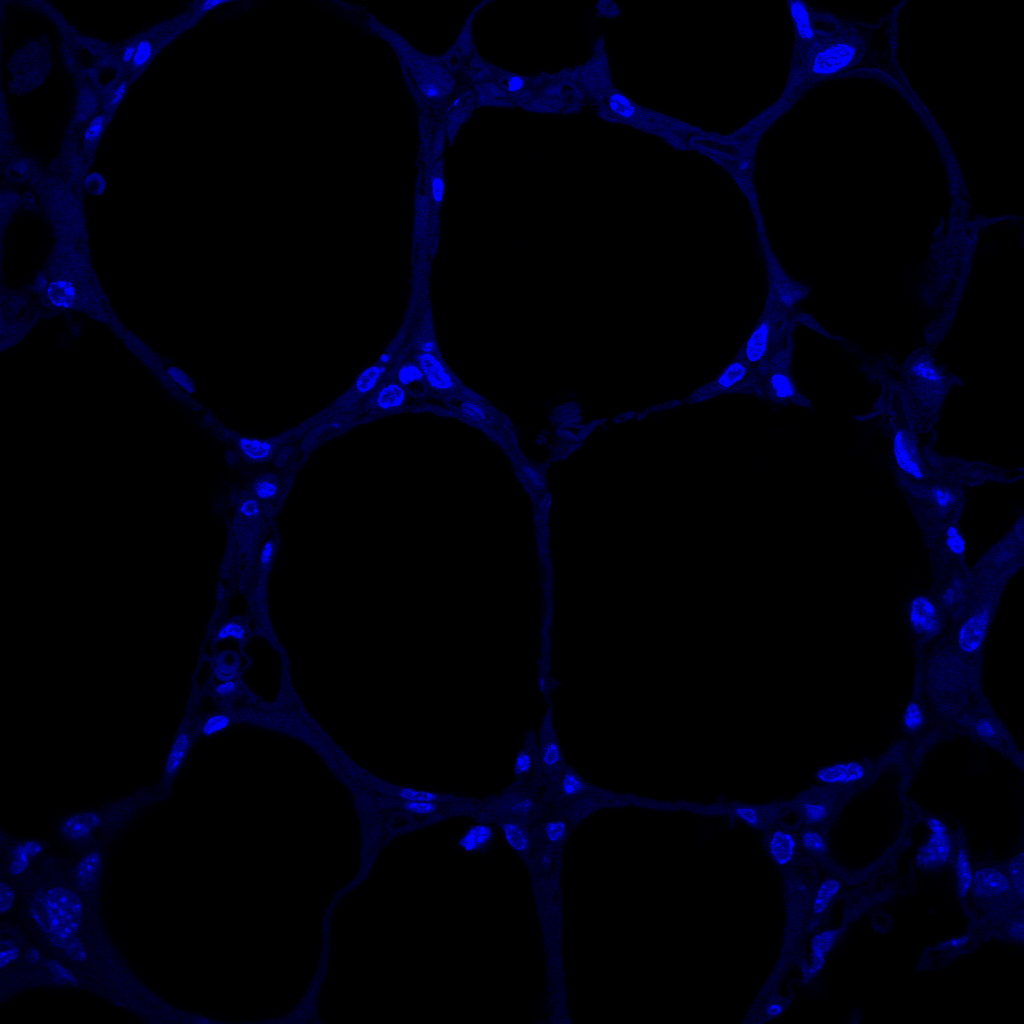

Supplement: Figure 3—source data 2. [file elife-72182-fig3-data2.zip › Adipose tissues/cela bip adi 2 (dapi).tif]

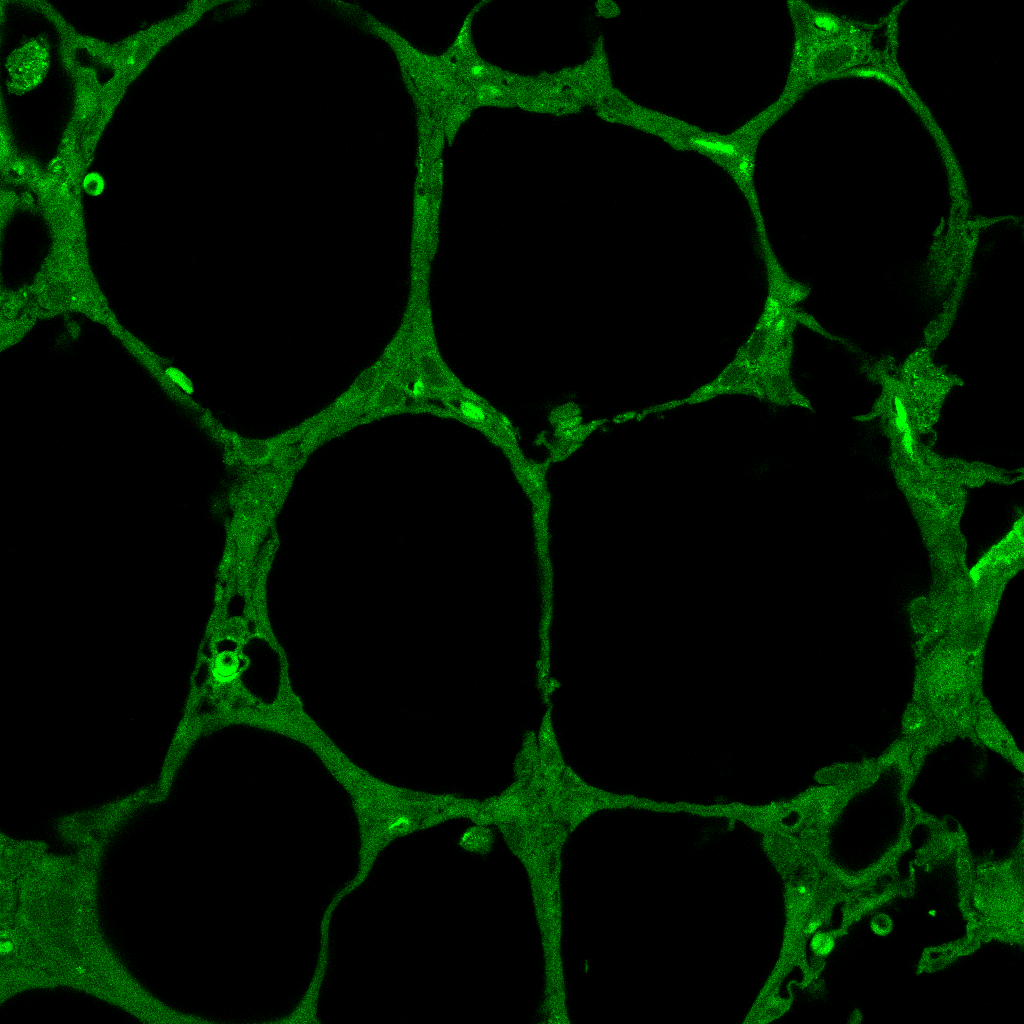

Supplement: Figure 3—source data 2. [file elife-72182-fig3-data2.zip › Adipose tissues/cela bip adi 2 (gr).tif]

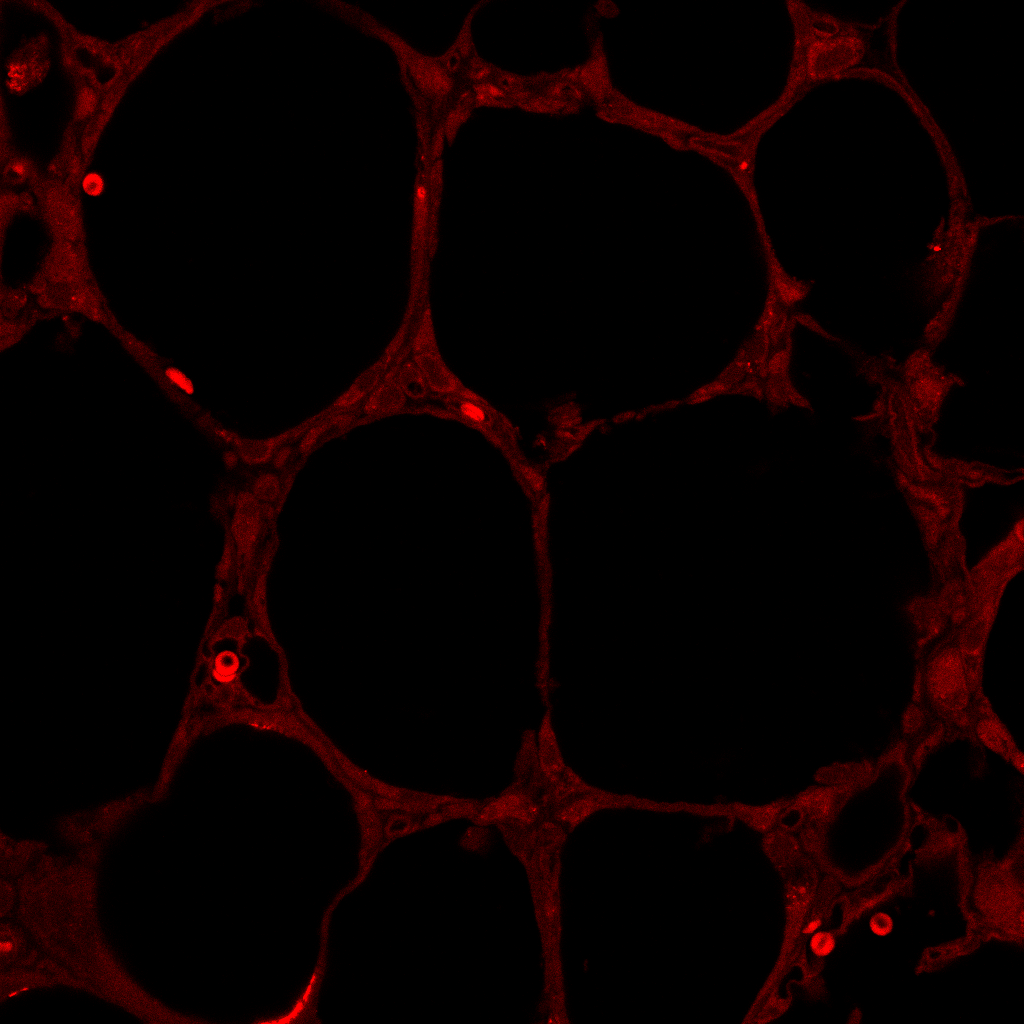

Supplement: Figure 3—source data 2. [file elife-72182-fig3-data2.zip › Adipose tissues/cela bip adi 2 (red).tif]

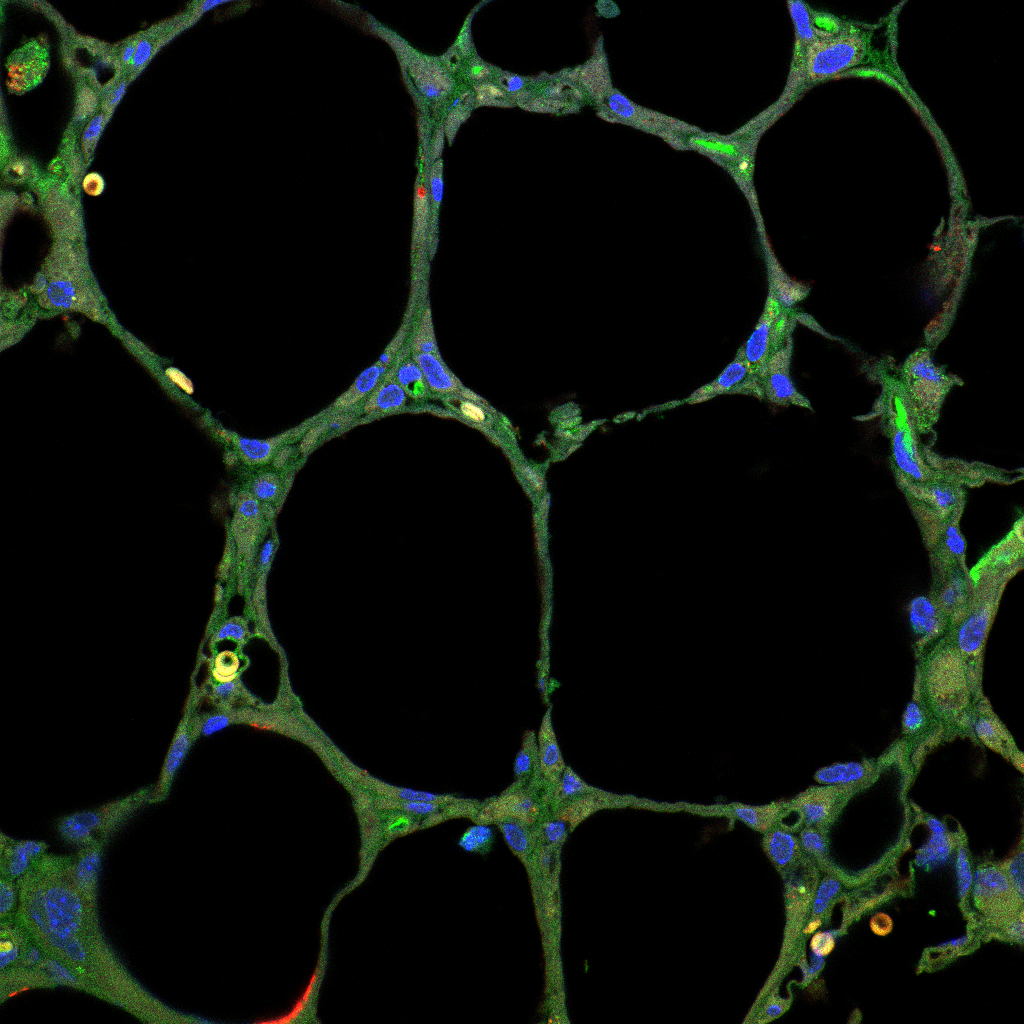

Supplement: Figure 3—source data 2. [file elife-72182-fig3-data2.zip › Adipose tissues/cela bip adi 2.tif]

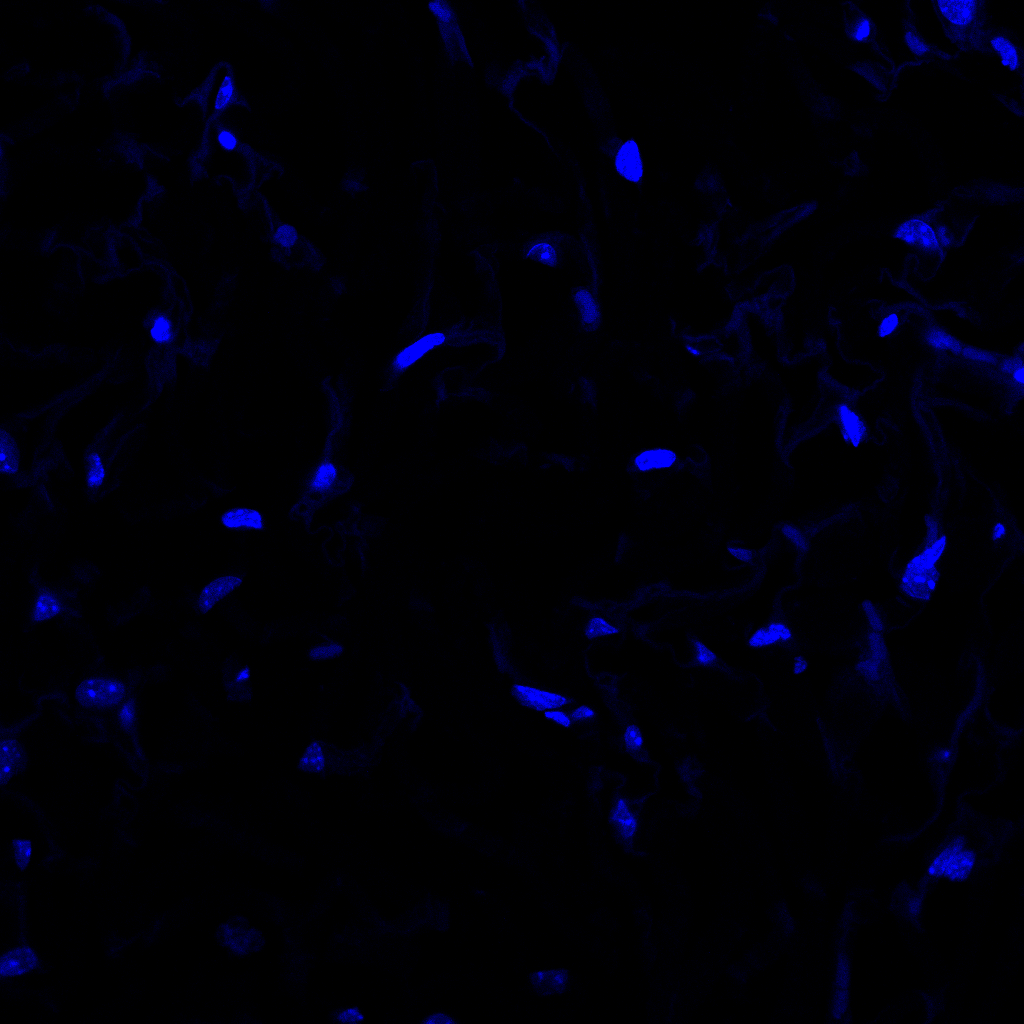

Supplement: Figure 3—source data 2. [file elife-72182-fig3-data2.zip › Adipose tissues/ctrl bip adi (dapi).tif]

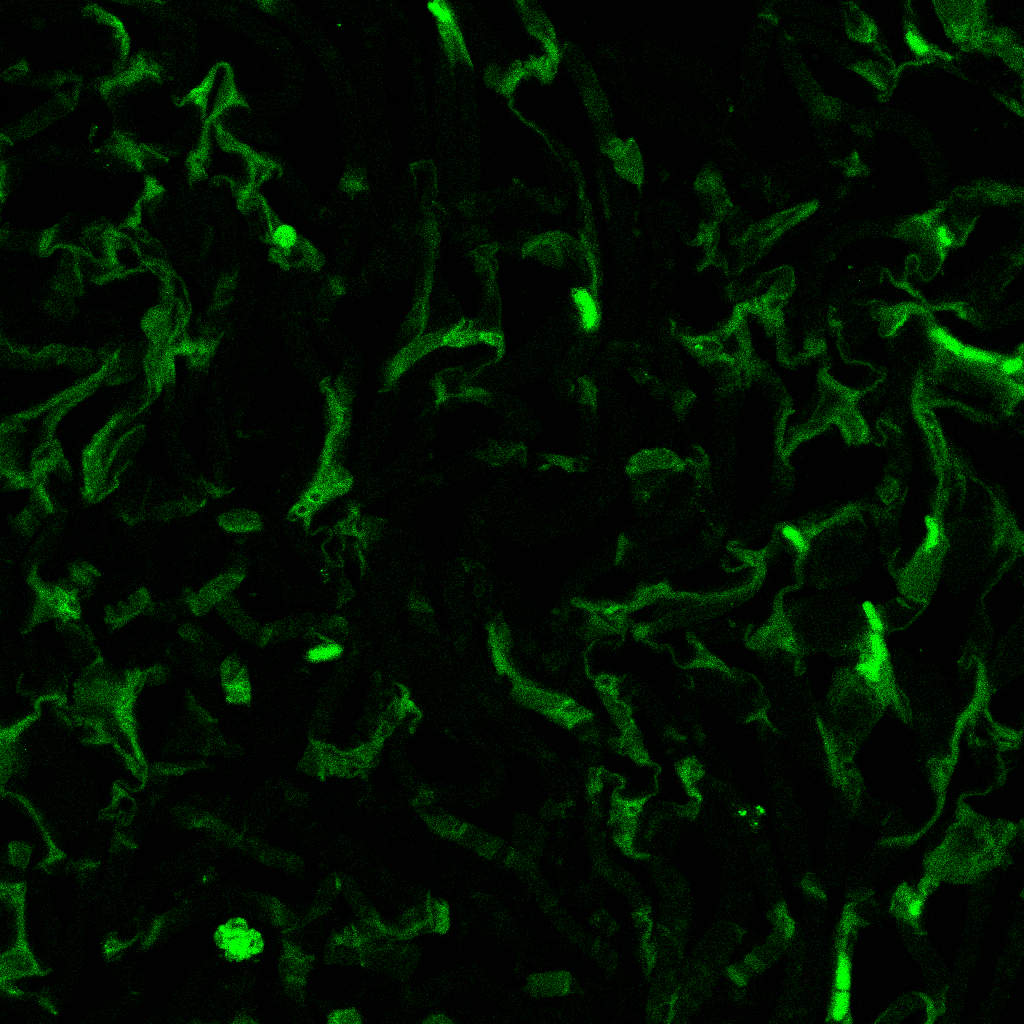

Supplement: Figure 3—source data 2. [file elife-72182-fig3-data2.zip › Adipose tissues/ctrl bip adi (green).tif]

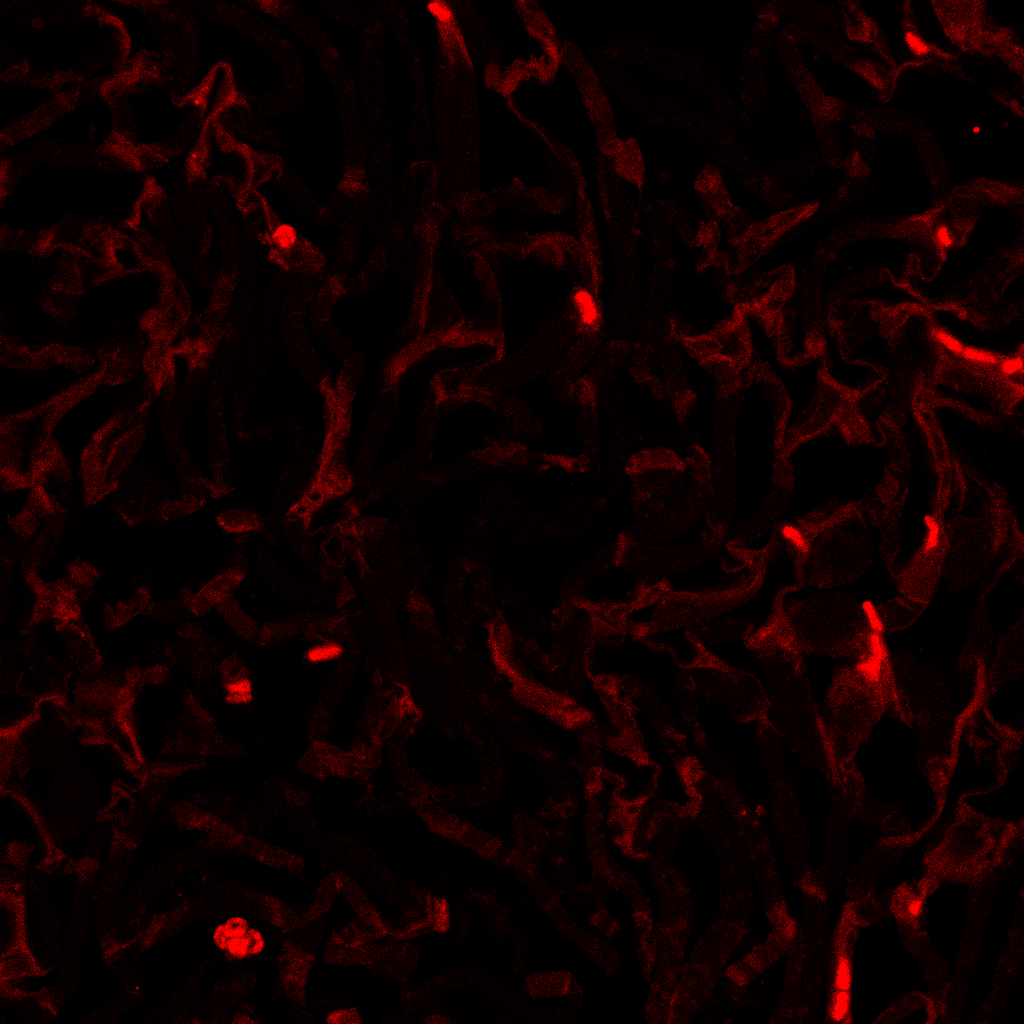

Supplement: Figure 3—source data 2. [file elife-72182-fig3-data2.zip › Adipose tissues/ctrl bip adi (red).tif]

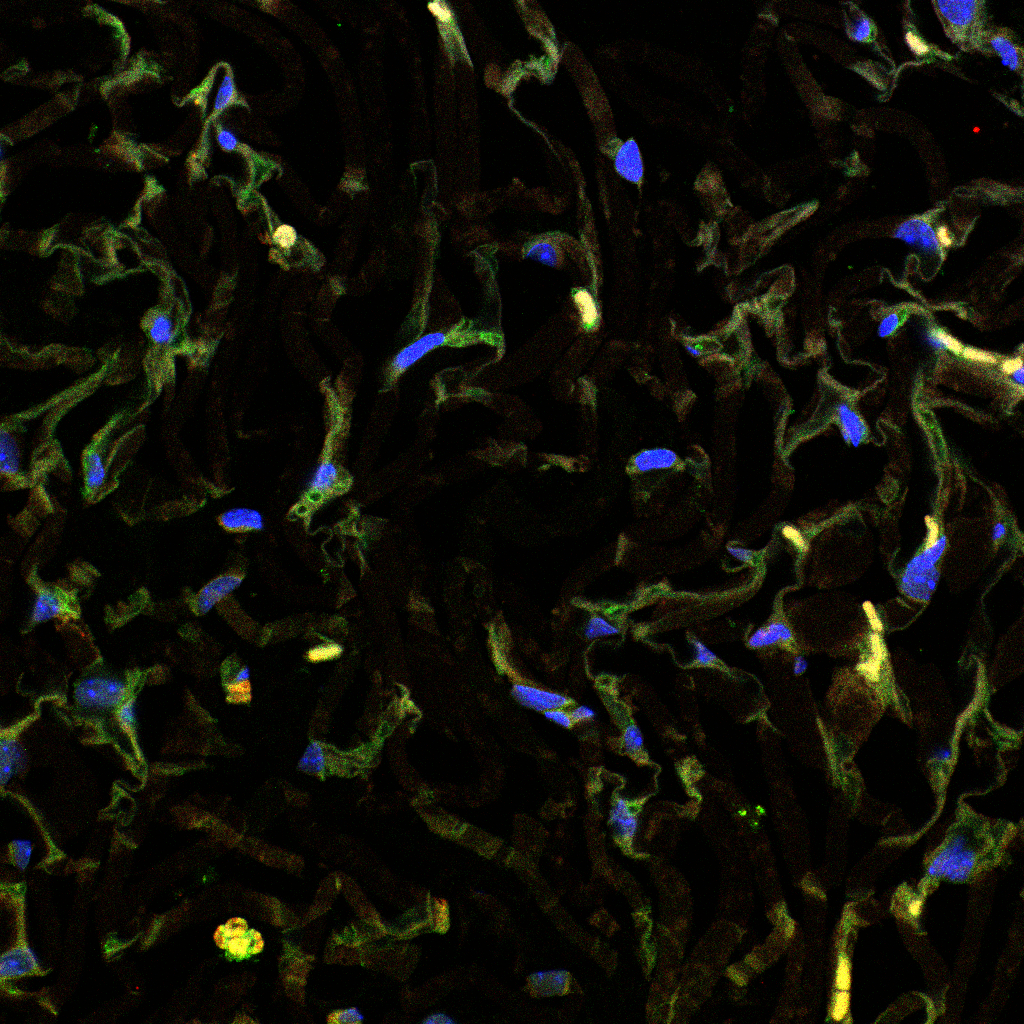

Supplement: Figure 3—source data 2. [file elife-72182-fig3-data2.zip › Adipose tissues/ctrl bip adi 1.tif]

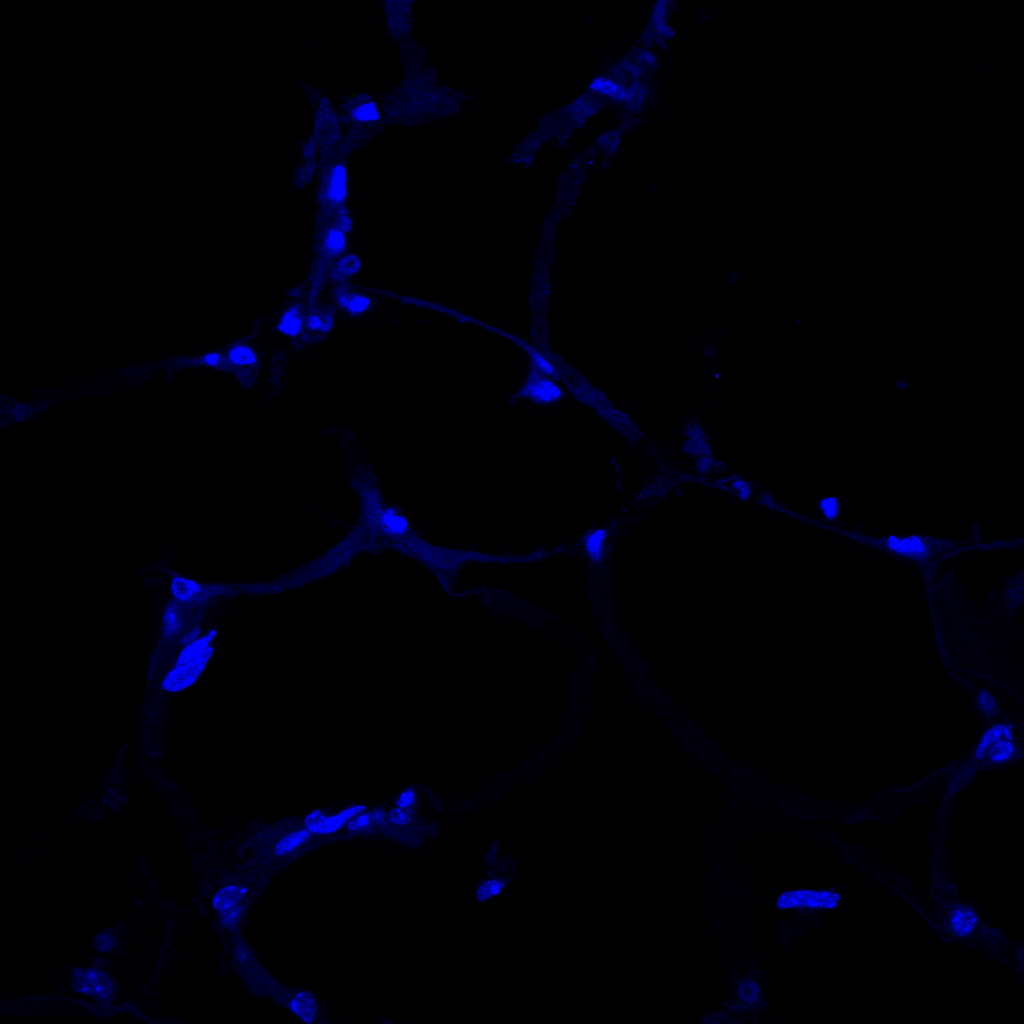

Supplement: Figure 3—source data 2. [file elife-72182-fig3-data2.zip › Adipose tissues/hfd bip adi 2 (dapi).tif]

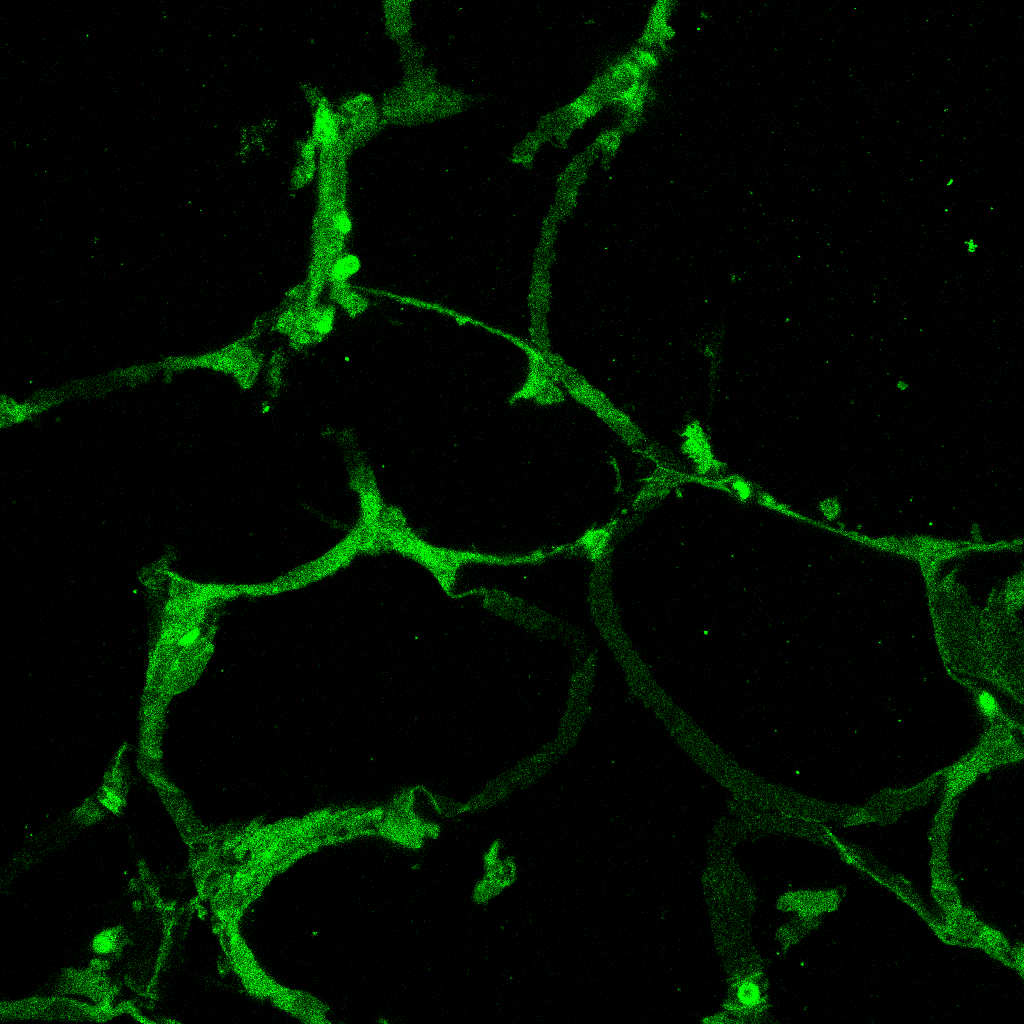

Supplement: Figure 3—source data 2. [file elife-72182-fig3-data2.zip › Adipose tissues/hfd bip adi 2 (green).tif]

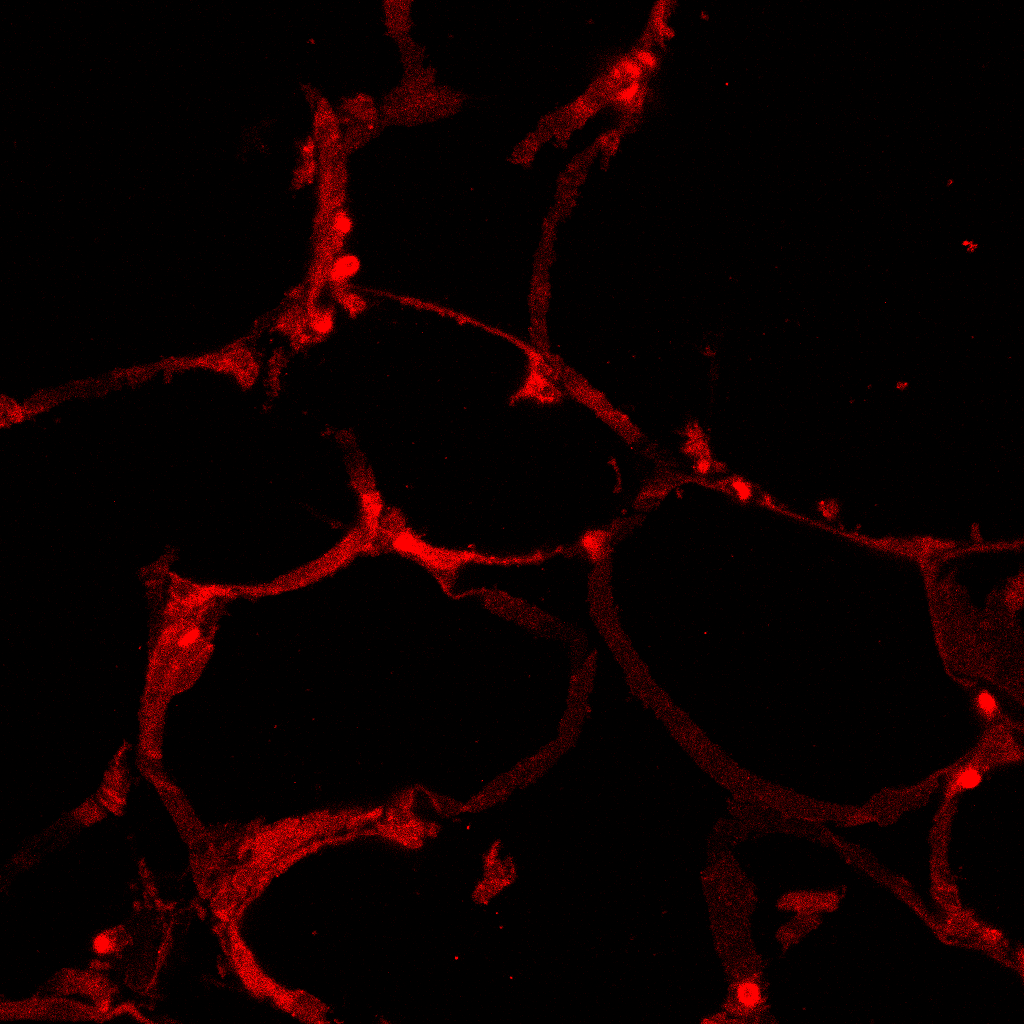

Supplement: Figure 3—source data 2. [file elife-72182-fig3-data2.zip › Adipose tissues/hfd bip adi 2 (red).tif]

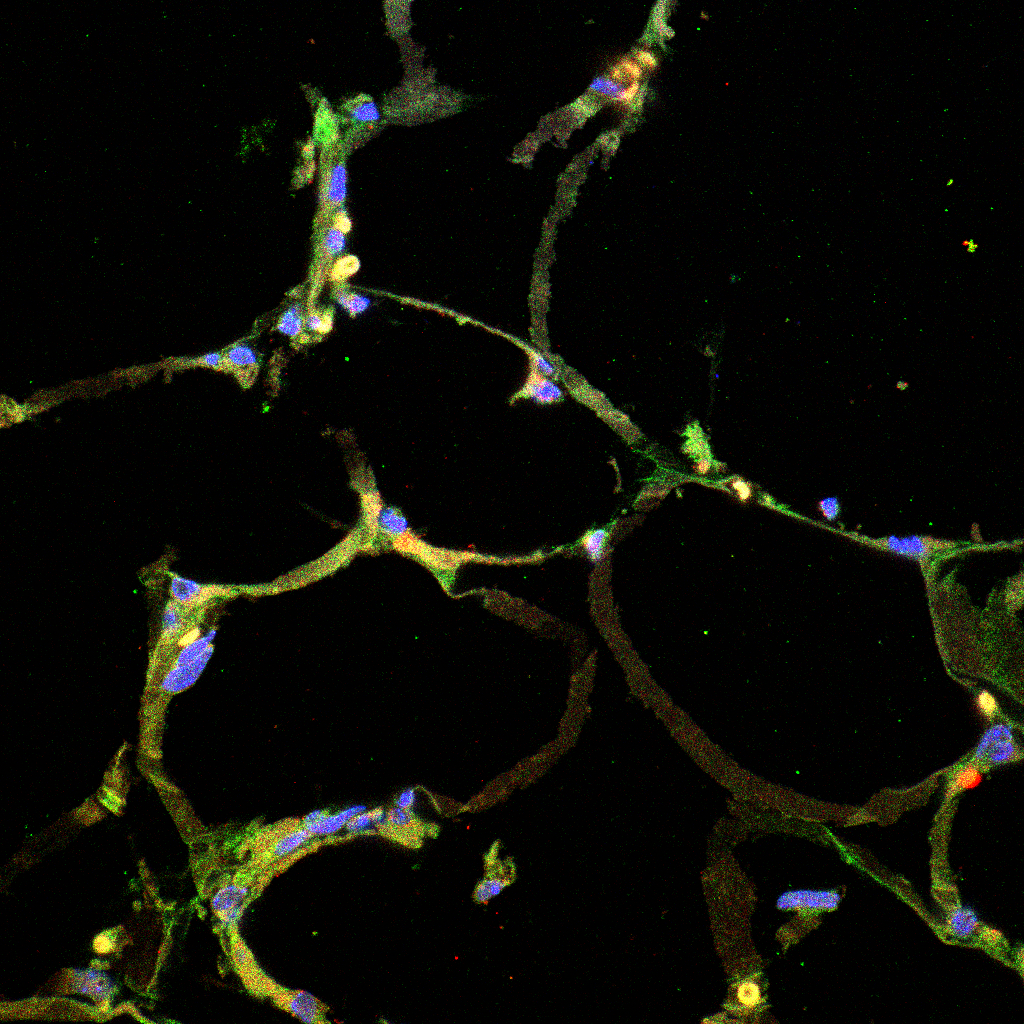

Supplement: Figure 3—source data 2. [file elife-72182-fig3-data2.zip › Adipose tissues/hfd bip adi 2.tif]

## Slide 1
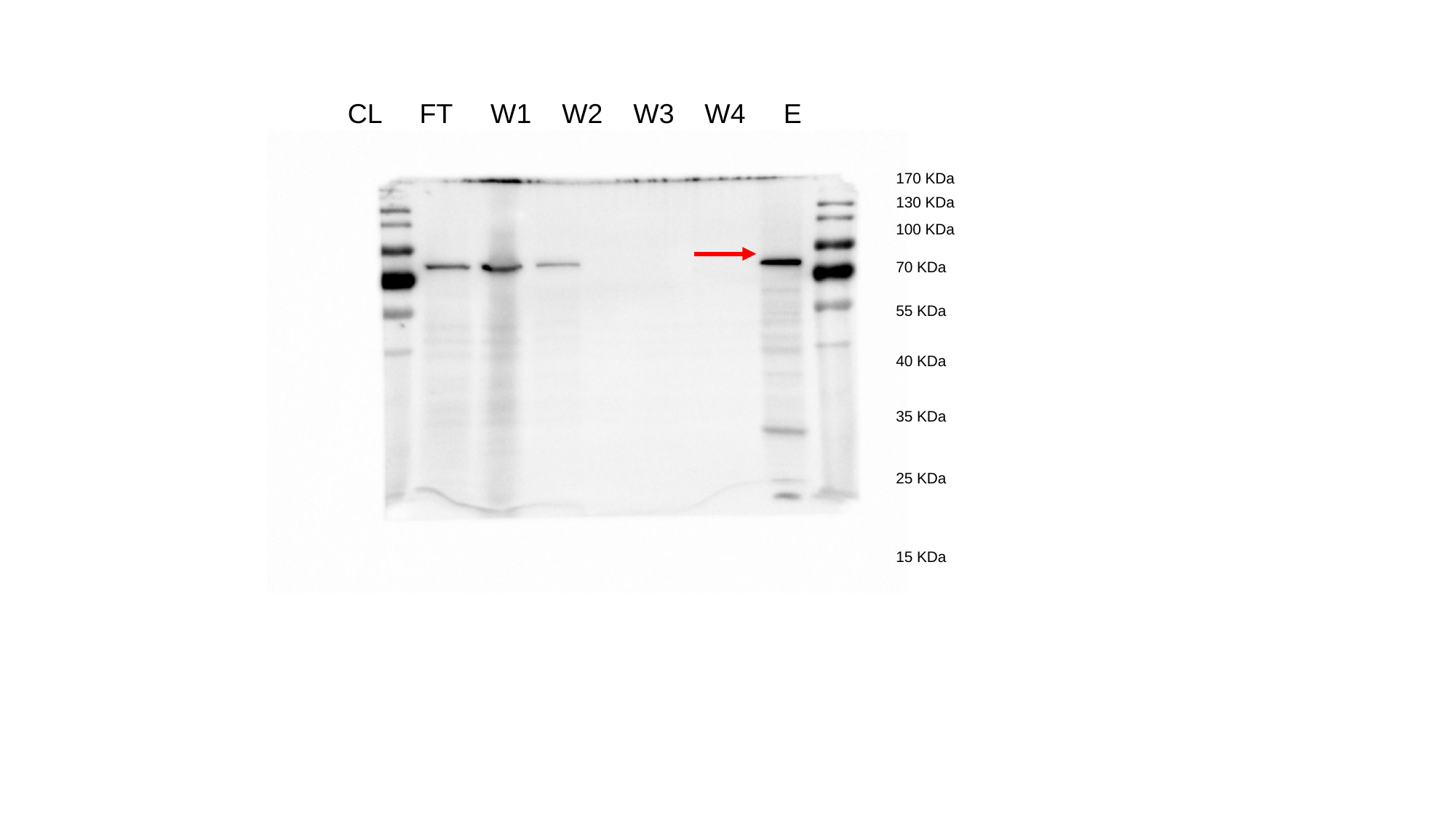

CL FT W1 W2 W3 W4 E
170 KDa
130 KDa
100 KDa
70 KDa
55 KDa
40 KDa
35 KDa
25 KDa
15 KDa

Supplement: Figure 6—source data 1. [file elife-72182-fig6-data1.zip › Figure 6-source data1/Figure 6B.pptx]
