# Supplementary material for: Covalent inhibition of endoplasmic reticulum chaperone GRP78 disconnects the transduction of ER stress signals to inflammation and lipid accumulation in diet-induced obese mice
Source: eLife. 2022 Feb 9;11:e72182. doi: 10.7554/eLife.72182 (PMC8828050; doi:10.7554/eLife.72182)
Supplement: Figure 3—source data 3. [file elife-72182-fig3-data3.zip › Figure 3-source data3/Liver tissues/Liver-GRP78 WB.pptx]

## Slide 1
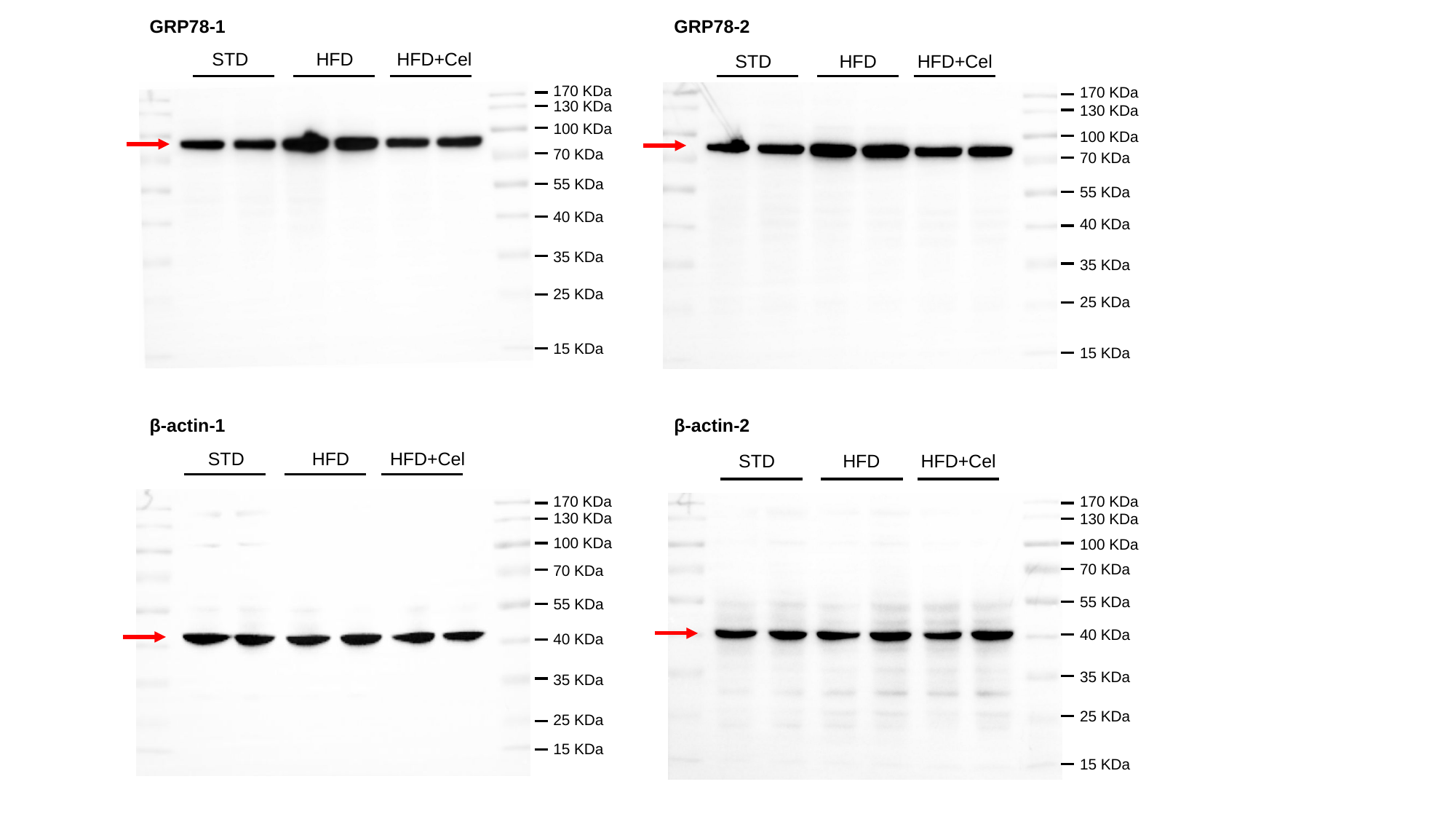

GRP78-1
GRP78-2
STD
 HFD
HFD+Cel
STD
 HFD
HFD+Cel
170 KDa
130 KDa
100 KDa
70 KDa
55 KDa
40 KDa
35 KDa
25 KDa
15 KDa
170 KDa
130 KDa
100 KDa
70 KDa
55 KDa
40 KDa
35 KDa
25 KDa
15 KDa
β-actin-1
β-actin-2
STD
 HFD
HFD+Cel
STD
 HFD
HFD+Cel
170 KDa
130 KDa
100 KDa
70 KDa
55 KDa
40 KDa
35 KDa
25 KDa
15 KDa
170 KDa
130 KDa
100 KDa
70 KDa
55 KDa
40 KDa
35 KDa
25 KDa
15 KDa
