# Supplementary material for: Covalent inhibition of endoplasmic reticulum chaperone GRP78 disconnects the transduction of ER stress signals to inflammation and lipid accumulation in diet-induced obese mice
Source: eLife. 2022 Feb 9;11:e72182. doi: 10.7554/eLife.72182 (PMC8828050; doi:10.7554/eLife.72182)
Supplement: Figure 7—source data 2. [file elife-72182-fig7-data2.zip › Figure 7-source data2/Figure 7D.pptx]

## Slide 1
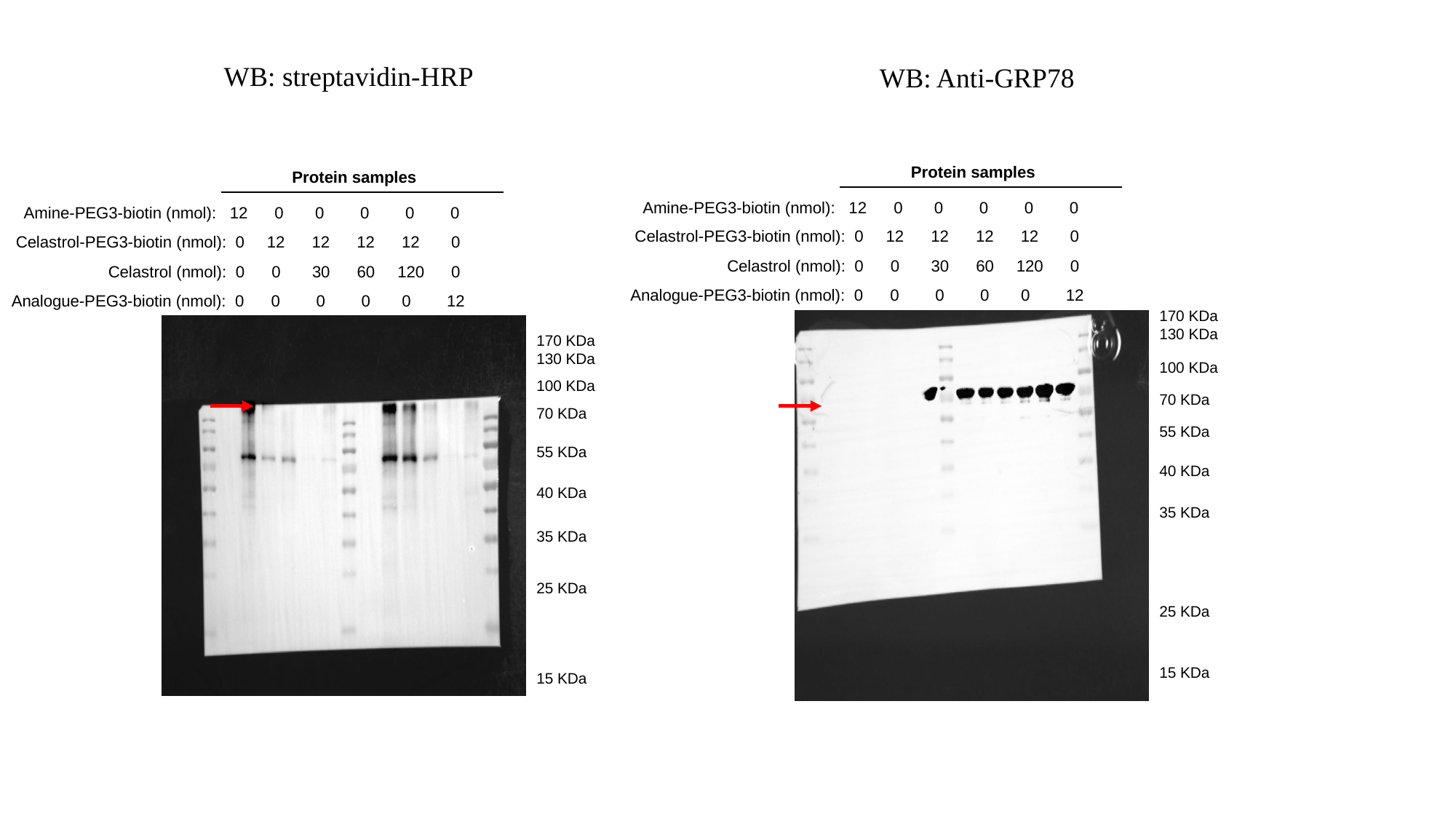

WB: streptavidin-HRP
 WB: Anti-GRP78
Protein samples
Amine-PEG3-biotin (nmol): 12 0 0 0 0 0
Celastrol-PEG3-biotin (nmol): 0 12 12 12 12 0
Celastrol (nmol): 0 0 30 60 120 0
Analogue-PEG3-biotin (nmol): 0 0 0 0 0 12
Protein samples
Amine-PEG3-biotin (nmol): 12 0 0 0 0 0
Celastrol-PEG3-biotin (nmol): 0 12 12 12 12 0
Celastrol (nmol): 0 0 30 60 120 0
Analogue-PEG3-biotin (nmol): 0 0 0 0 0 12
170 KDa
130 KDa
100 KDa
70 KDa
55 KDa
40 KDa
35 KDa
25 KDa
15 KDa
170 KDa
130 KDa
100 KDa
70 KDa
55 KDa
40 KDa
35 KDa
25 KDa
15 KDa
