# Supplementary material for: Covalent inhibition of endoplasmic reticulum chaperone GRP78 disconnects the transduction of ER stress signals to inflammation and lipid accumulation in diet-induced obese mice
Source: eLife. 2022 Feb 9;11:e72182. doi: 10.7554/eLife.72182 (PMC8828050; doi:10.7554/eLife.72182)
Supplement: Figure 7—source data 3. [file elife-72182-fig7-data3.pptx]

## Slide 1
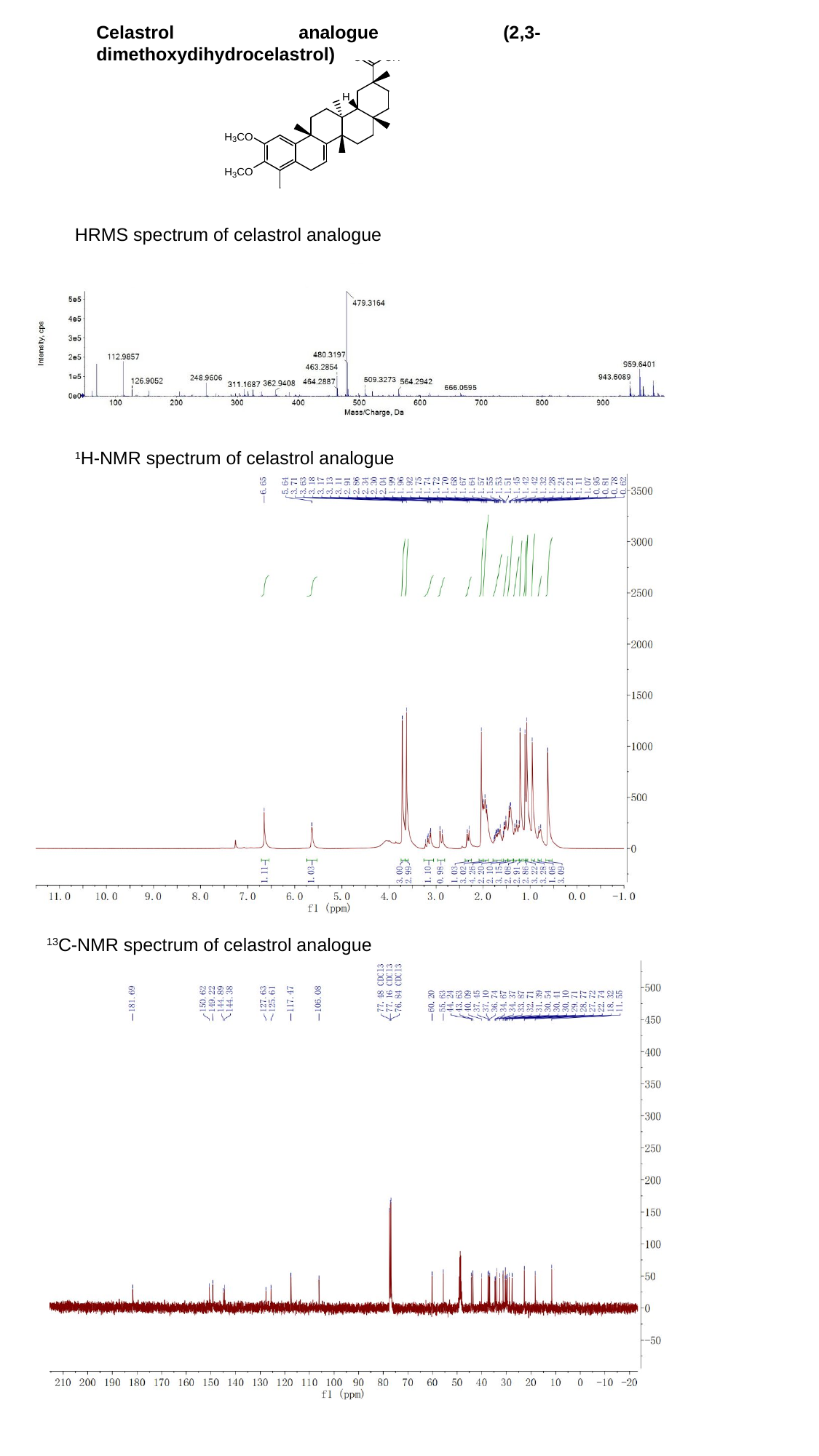

Celastrol analogue (2,3-dimethoxydihydrocelastrol)
HRMS spectrum of celastrol analogue
1H-NMR spectrum of celastrol analogue
13C-NMR spectrum of celastrol analogue
