# Supplementary material for: Covalent inhibition of endoplasmic reticulum chaperone GRP78 disconnects the transduction of ER stress signals to inflammation and lipid accumulation in diet-induced obese mice
Source: eLife. 2022 Feb 9;11:e72182. doi: 10.7554/eLife.72182 (PMC8828050; doi:10.7554/eLife.72182)
Supplement: Figure 7—source data 4. [file elife-72182-fig7-data4.pptx]

## Slide 1
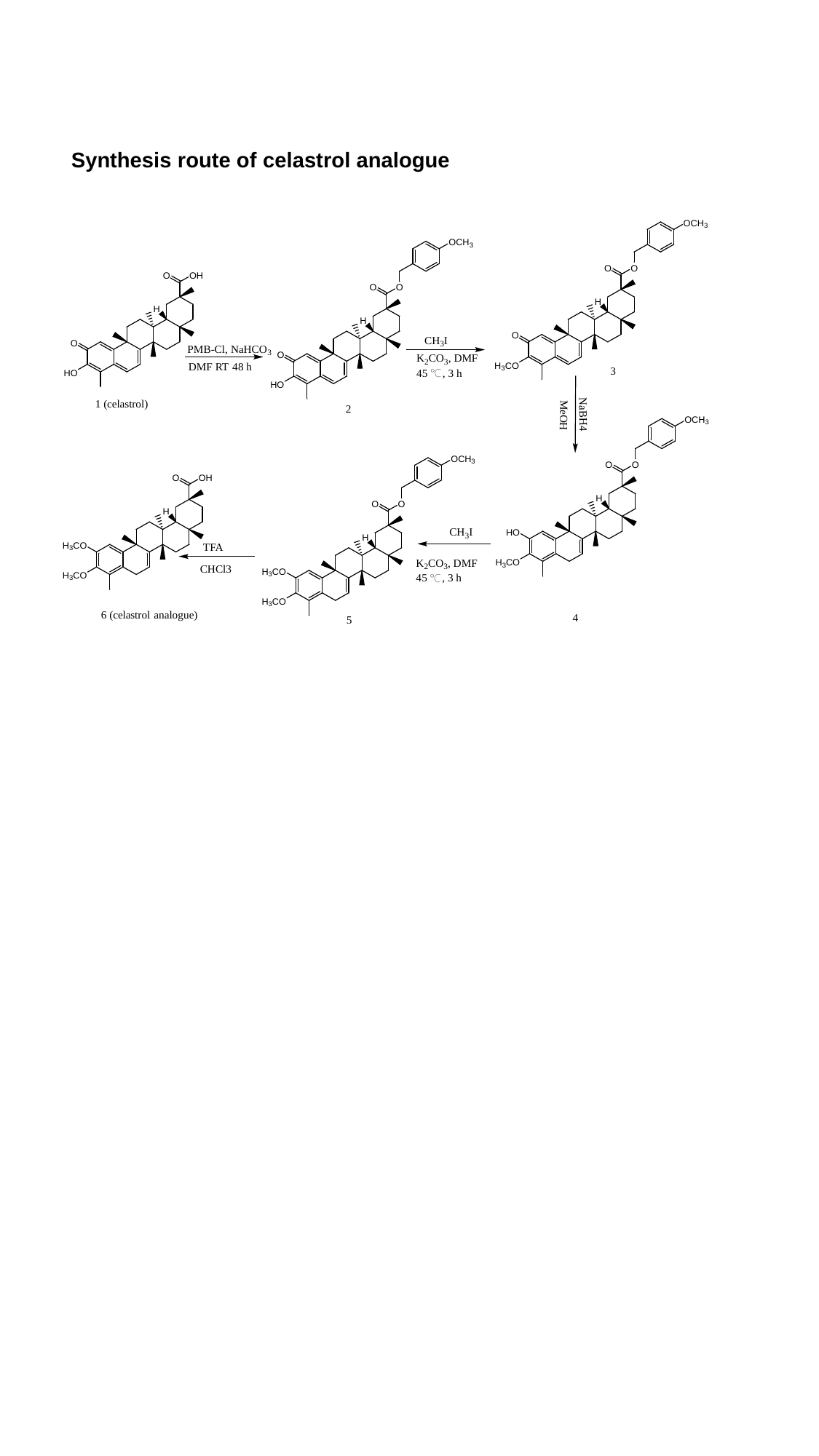

Synthesis route of celastrol analogue

## Slide 2
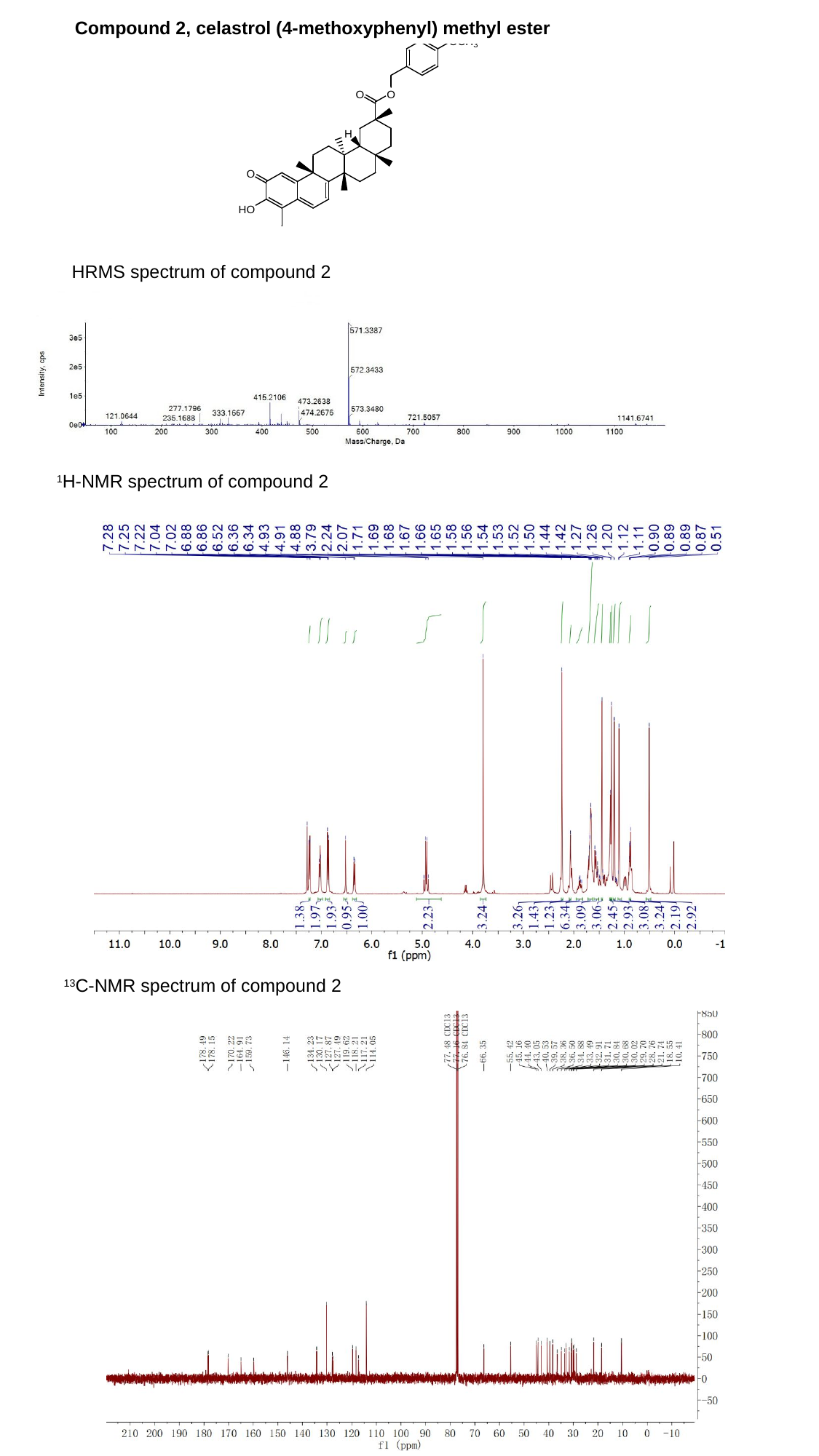

Compound 2, celastrol (4-methoxyphenyl) methyl ester
HRMS spectrum of compound 2
1H-NMR spectrum of compound 2
13C-NMR spectrum of compound 2

## Slide 3
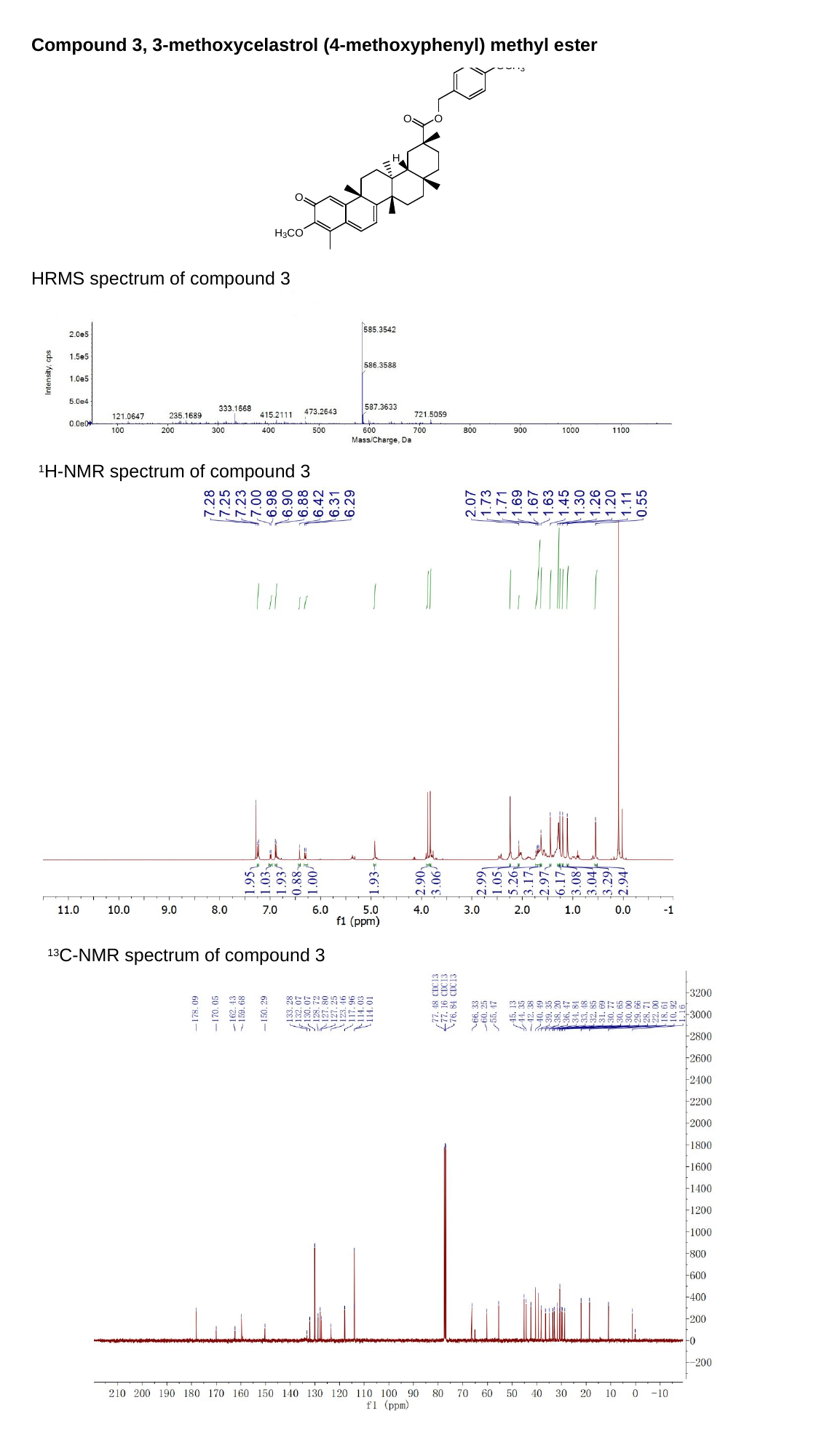

Compound 3, 3-methoxycelastrol (4-methoxyphenyl) methyl ester
HRMS spectrum of compound 3
1H-NMR spectrum of compound 3
13C-NMR spectrum of compound 3

## Slide 4
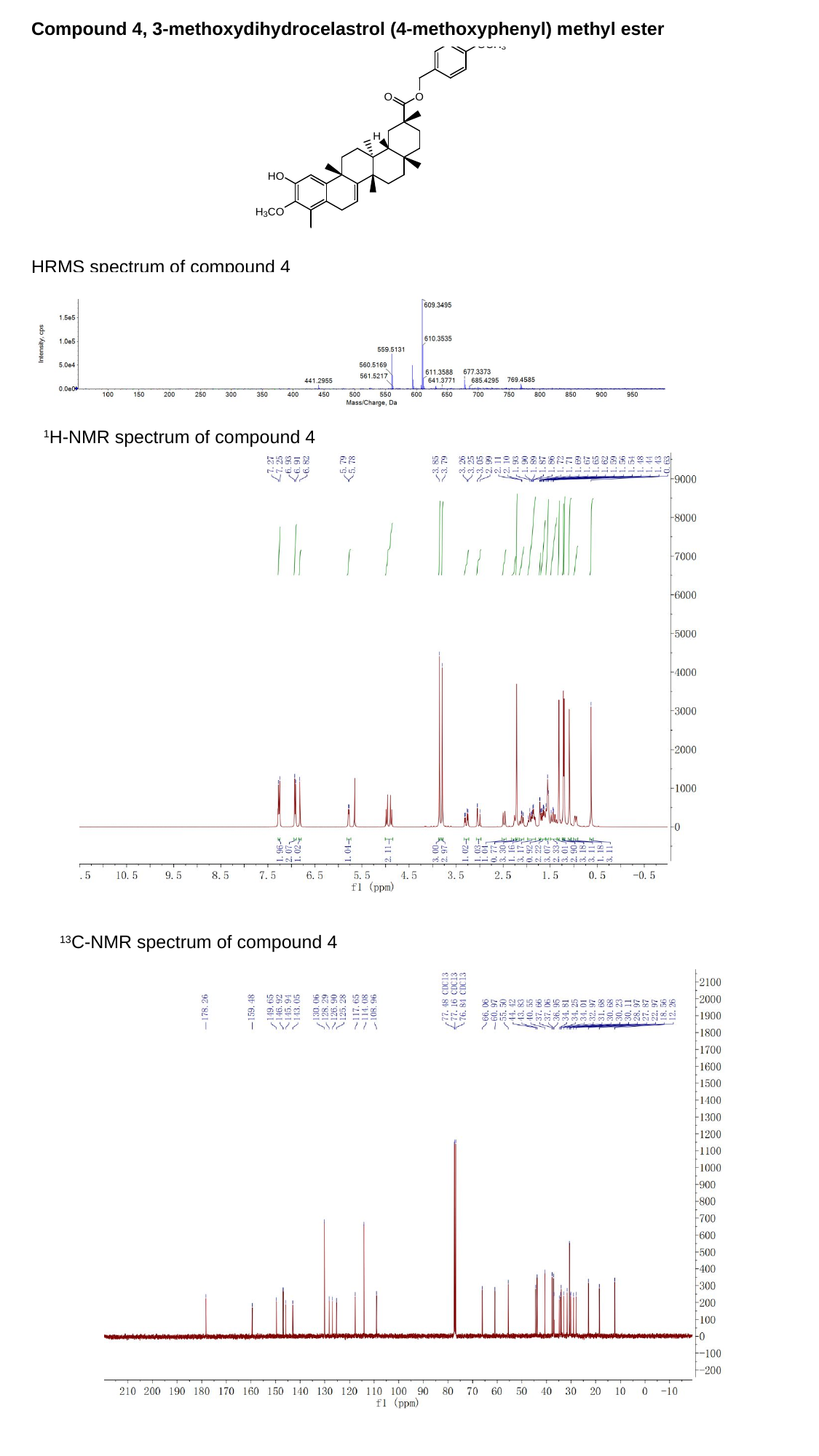

Compound 4, 3-methoxydihydrocelastrol (4-methoxyphenyl) methyl ester
HRMS spectrum of compound 4
1H-NMR spectrum of compound 4
13C-NMR spectrum of compound 4

## Slide 5
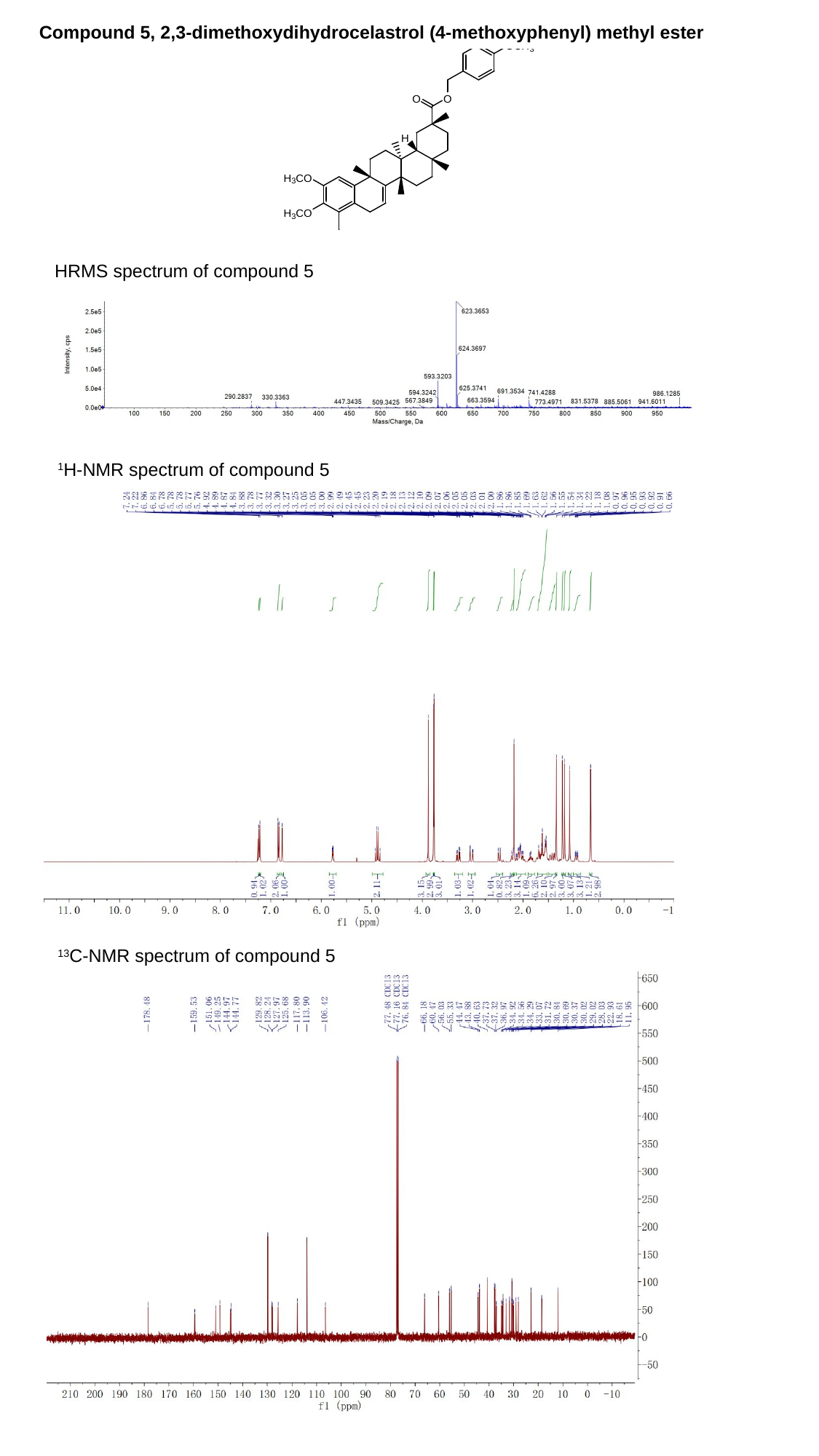

Compound 5, 2,3-dimethoxydihydrocelastrol (4-methoxyphenyl) methyl ester
HRMS spectrum of compound 5
1H-NMR spectrum of compound 5
13C-NMR spectrum of compound 5
