# Supplementary material for: Covalent inhibition of endoplasmic reticulum chaperone GRP78 disconnects the transduction of ER stress signals to inflammation and lipid accumulation in diet-induced obese mice
Source: eLife. 2022 Feb 9;11:e72182. doi: 10.7554/eLife.72182 (PMC8828050; doi:10.7554/eLife.72182)
Supplement: Figure 7—source data 5. [file elife-72182-fig7-data5.pptx]

## Slide 1
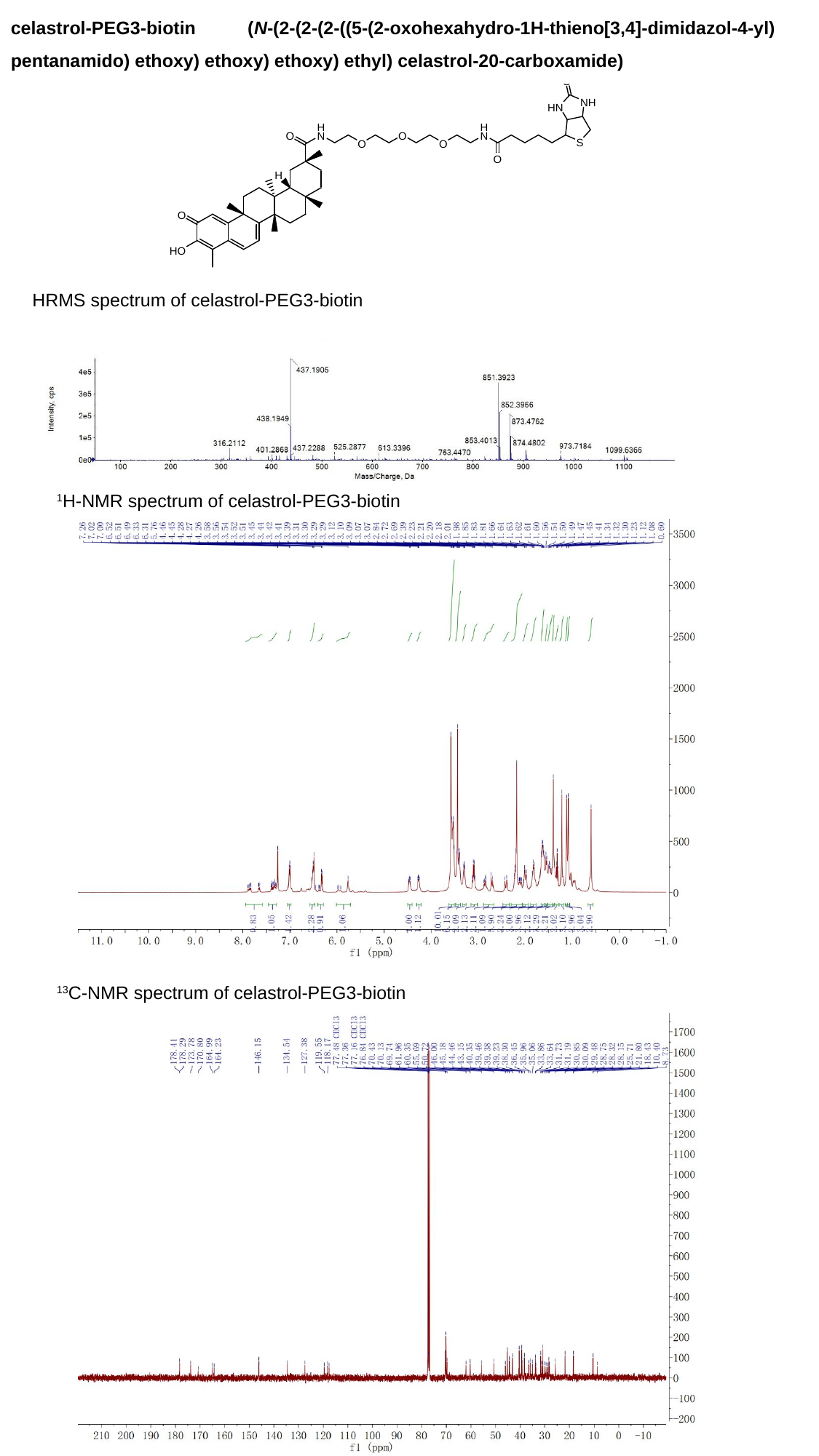

celastrol-PEG3-biotin (N-(2-(2-(2-((5-(2-oxohexahydro-1H-thieno[3,4]-dimidazol-4-yl) pentanamido) ethoxy) ethoxy) ethoxy) ethyl) celastrol-20-carboxamide)
HRMS spectrum of celastrol-PEG3-biotin
1H-NMR spectrum of celastrol-PEG3-biotin
13C-NMR spectrum of celastrol-PEG3-biotin
